# Neuroimaging studies of GABA in schizophrenia: a systematic review with meta-analysis

**DOI:** 10.1038/tp.2017.124

**Published:** 2017-06-06

**Authors:** A Egerton, G Modinos, D Ferrera, P McGuire

**Affiliations:** 1 Department of Psychosis Studies, Institute of Psychiatry, Psychology and Neuroscience, King’s College London, London, UK; 2 Serviço de Psiquiatria e Saúde Mental, Departamento de Neurociências e Saúde Mental, Hospital de Santa Maria EPE, Lisboa, Portugal

## Abstract

Data from animal models and from postmortem studies suggest that schizophrenia is associated with brain GABAergic dysfunction. The extent to which this is reflected in data from *in vivo* studies of GABA function in schizophrenia is unclear. The Medline database was searched to identify articles published until 21 October 2016. The search terms included GABA, proton magnetic resonance spectroscopy (^1^H-MRS), positron emission tomography (PET), single photon emission computed tomography (SPECT), schizophrenia and psychosis. Sixteen GABA ^1^H-MRS studies (538 controls, 526 patients) and seven PET/SPECT studies of GABA_A_/benzodiazepine receptor (GABA_A_/BZR) availability (118 controls, 113 patients) were identified. Meta-analyses of ^1^H-MRS GABA in the medial prefrontal cortex (mPFC), parietal/occipital cortex (POC) and striatum did not show significant group differences (mFC: *g*=−0.3, 409 patients, 495 controls, 95% confidence interval (CI): −0.6 to 0.1; POC: *g*=−0.3, 139 patients, 111 controls, 95% CI: −0.9 to 0.3; striatum: *g*=−0.004, 123 patients, 95 controls, 95% CI: −0.7 to 0.7). Heterogeneity across studies was high (*I*^2^>50%), and this was not explained by subsequent moderator or meta-regression analyses. There were insufficient PET/SPECT receptor availability studies for meta-analyses, but a systematic review did not suggest replicable group differences in regional GABA_A_/BZR availability. The current literature does not reveal consistent alterations in *in vivo* GABA neuroimaging measures in schizophrenia, as might be hypothesized from animal models and postmortem data. The analysis highlights the need for further GABA neuroimaging studies with improved methodology and addressing potential sources of heterogeneity.

## Introduction

One of the most consistent findings from postmortem studies in schizophrenia is a reduction in the GABA-synthesizing enzyme, GAD67 mRNA and protein.^1, 2, 3^ Expression of GAD67 is activity regulated^4, 5^ and GAD67 is responsible for over 90% of all (cytosolic) GABA production.^6^ In contrast to GAD67, inconsistent findings in schizophrenia are reported for the GAD65 isoform,^7, 8, 9, 10^ which is involved in vesicular, synaptic GABA production during intense periods of neural activity.^11, 12^ The potential effects of a reduction in GAD67 on cortical excitatory/inhibitory networks is a key component in some neurobiological models of schizophrenia.^13^ In particular, GABA dysfunction is thought to lead to the disinhibition of glutamatergic pyramidal neurons and a loss of synchronous cortical activity.^14, 15^ Postmortem studies also suggest that schizophrenia is associated with dysfunctional GABA signalling at the postsynaptic receptor level. Receptor autoradiography using ^3^H-muscimol, an agonist at the GABA binding site on the GABA_A_/benzodiazepine receptor (GABA_A_/BZR) complex, has consistently shown an increase in binding density in the prefrontal, cingulate and temporal cortices and caudate nucleus.^16, 17, 18, 19, 20, 21, 22^ In contrast, density of binding to the BZ binding site of the GABA_A_/BZR complex has been found unaltered, increased or decreased postmortem.^22, 23, 24, 25, 26, 27^ Postmortem investigations of GABA_A_ α subunit expression have found reductions in α1 (refs 28, 29) and increases in α2 (refs 29, 30) expression in schizophrenia, but inconsistent results for the α5 subunit.^29, 31, 32^

GABA function in schizophrenia can be assessed *in vivo* using neuroimaging techniques. Proton magnetic resonance spectroscopy (^1^H-MRS) optimized for GABA detection can measure GABA concentrations within a voxel of interest. This approach measures total (intracellular and extracellular) GABA and macromolecules (denoted GABA+) across all tissue content in a relatively large voxel. An alternative neuroimaging approach is to use positron emission tomography (PET) or single photon emission computed tomography (SPECT) in conjunction with specific radiotracers that bind to GABA or BZ receptors.^33^ However, all the PET/SPECT radiotracers currently available for human use bind to the BZ rather than to the GABA_A_ site of GABA_A_/BZ receptors. The PET/SPECT radiotracers iomazenil and flumazenil have limited subunit selectivity, binding GABA_A_/BZ receptors containing α1, α2, α3 and α6 subunits, whereas Ro15-4513 has more selectivity for α1 and α5.^34^

Neuroimaging of GABA function is potentially important because several hypotheses around the role of GABA dysfunction in schizophrenia can only be tested *in vivo*. Evidence that GABA dysfunction has a role in the pathophysiology of schizophrenia has also led to interest in the therapeutic potential of pharmacological compounds that act on GABA function, and data from animal studies suggest that administration of benzodiazepines can prevent the development of neuroanatomical and neurophysiological abnormalities associated with schizophrenia.^35, 36^

Although there have been several neuroimaging studies of GABA in schizophrenia, the nature of GABAergic abnormalities in schizophrenia *in vivo* remains unclear. The present study aims to address this issue by conducting a systematic review and a meta-analysis of ^1^H-MRS and PET/SPECT studies of GABA in schizophrenia. In our primary analyses as well as studies of patients with schizophrenia, we included studies of subjects at high clinical or genetic risk for the disorder, as GABAergic dysfunction may be a ‘trait’ characteristic, arising through the influences of genetic variation during development.^37^ The potential influence of clinical subgroups,^38, 39^ medication status,^38, 40^ symptom severity,^38, 40, 41^ age^42, 43^ and gender^44^ were investigated using subsequent moderator analyses and meta-regression.

## Materials and methods

### Study selection

The meta-analysis and systematic review was performed in accordance with the guidelines of the PRISMA group.^45^ The Medline electronic database was searched to identify journal articles published from 1 January 1950 until 21 October 2016, using the following MeSH and freeform search terms: (‘GABA’) AND (‘MRS’ OR ‘spectroscopy’ OR ‘positron emission tomography’ OR ‘single photon emission tomography’ OR ‘single photon emission computed tomography’ OR ‘PET’ OR ‘SPET’ OR ‘SPECT’) AND (‘schizophrenia’ OR ‘psychosis’ OR ‘schizophreniform’ OR ‘psychosis risk’). Reference lists of the returned articles were hand searched for further relevant publications. Two authors independently performed the searches and identified articles for inclusion (AE and DF).

Inclusion required that articles were published in peer-reviewed journals in English or English translation. Inclusion also required that articles reported GABA measures *in vivo,* in a group with clinical diagnosis of schizophrenia, schizoaffective disorder or first episode psychosis, or a group at clinical or genetic risk for schizophrenia, compared with a healthy volunteer (control) group. 1H-MRS studies were excluded if they reported the GABA signal only as the combined signal with glutamate (Glx). PET/SPECT studies were excluded if they investigated translocator protein, which mediates various mitochondrial functions and was previously described as the peripheral benzodiazepine receptor.^46^ Where articles reported overlapping samples, only data from the article reporting the largest sample was included.

### Outcome measures

The primary outcome measure was the control and patient mean and standard deviation (s.d.) ^1^H-MRS GABA+ concentration in each voxel, or GABA_A_/BZR availability in each region of interest. Where these values were not reported in the published article, the authors were contacted or values were estimated from figures using a freely available ruler for Mac OS X (http://www.pascal.com/software/freeruler/). Where values were reported in each hemisphere separately, the mean of these values was calculated. For ^1^H-MRS studies, due to partially overlapping voxel locations and to provide sufficient data for meta-analysis, data were combined into the medial frontal cortex (mFC), parietal and occipital cortices and striatum. All the variables were extracted independently by two authors (AE and DF) and cross-checked for accuracy.

### Meta-analysis

Inclusion in the meta-analyses required availability of data in a given brain region from five or more studies.^47^ Where there were insufficient data for meta-analysis, the findings were summarized. Where articles included more than one patient or control group, these groups were entered separately in the analyses. For each variable, the effect size statistic Hedges’ *g* was calculated. Hedges’ *g* is the Cohen’s effect size incorporating a correction for bias from small sample sizes.^48^

The meta-analysis for each variable was performed using STATA/IC, version 14, using the METAN command (StataCorp LP, College Station, TX, USA). A random-effects inverse-weighted variance model^49^ was used to calculate the pooled effect size to adjust for study heterogeneity. Significance was assessed using two-sided 95% confidence intervals.

Heterogeneity was measured using the *I*^2^ value, which indicates the percentage variance due to heterogeneity between studies compared with chance.^50^ Where *I*^2^ values indicated substantial heterogeneity (*I*^2^>50%), potential sources of heterogeneity were investigated by using sensitivity analysis to assess potential influences of single studies, and Egger’s test^51^ to investigate potential publication bias.

### Moderator analyses

**Figure 1:**
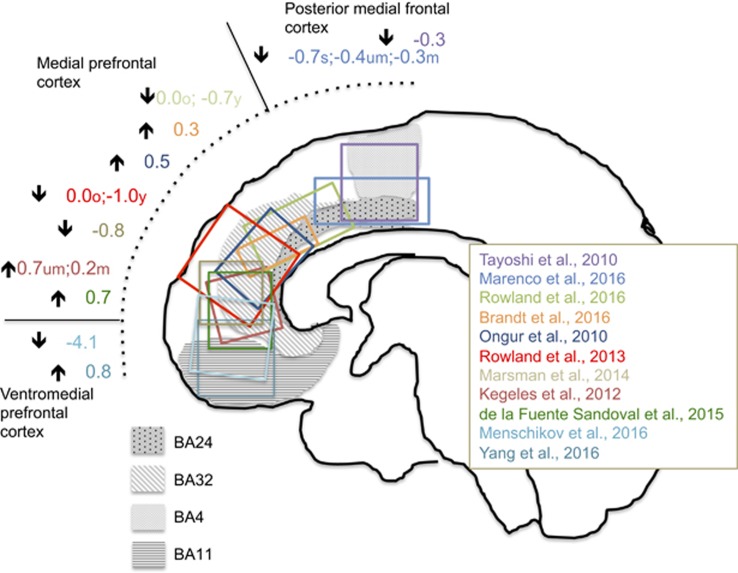
Voxel locations in medial frontal cortex GABA ^1^H-MRS studies. Numbers provide effect sizes (Hedge’s *g*) for the difference in ^1^H-MRS GABA level between patients and control participants for each study. Negative effect sizes indicate lower GABA in patients; positive effect sizes indicate lower GABA in controls. Subgroup membership was defined by voxel locations primarily in the Brodmann Areas (BA) BA4 and BA24 (posterior medial frontal cortex), BA24 and BA32 (medial prefrontal cortex) or BA24 and BA11 (ventromedial prefrontal cortex). ^1^H-MRS, proton magnetic resonance spectroscopy.

Potential influences of study characteristics were investigated using moderator analyses. Subgroup analyses investigated the following dichotomous characteristics of data sets: (1) clinical category of subjects (first episode psychosis or schizophrenia patients versus clinical risk or genetic risk groups); (2) explicitly stated absence of GABAergic (benzodiazepine or anticonvulsant) medication at the time of scanning; (3) presence (in >90% of the sample) or absence (in 100% of the sample) of antipsychotic medication at the time of imaging. For mFC GABA ^1^H-MRS studies, subgroup analyses additionally investigated potential influences of mFC voxel location (Figure 1).

Meta-regressions were conducted to explore potential influences of continuous variables relating to patient characteristics (age, percentage male in sample, illness duration, Positive and Negative Syndrome Scale total score), voxel grey matter content and publication year on GABA measures. Symptoms rated using the BPRS were converted to Positive and Negative Syndrome Scale scores using the established conversion scale of Leucht *et al.*^52^ Meta-regression analyses were performed in STATA/IC version 14 using the METAREG command, with Hedges’ *g* as the outcome variable. To reduce the likelihood of chance findings, both subgroup analyses and meta-regressions required a minimum of five data sets. In all cases, the threshold for statistical significance was *P*<0.05.

### Study quality and methodological characteristics

The methodological characteristics of ^1^H-MRS and PET/SPECT studies are summarized in Supplementary Tables 1 and 2, respectively. Although there are no established criteria for formal quality assessment of ^1^H-MRS and PET/SPECT studies, key factors that may impact on data quality are discussed in the Supplementary Information.

## Results

### ^1^H-MRS studies

Nineteen articles describing ^1^H-MRS studies of GABA+ in schizophrenia were identified (Supplementary Figure 1). Of these, the article by Chen *et al.*^53^ was excluded due to partial overlap with the larger sample reported in Kegeles *et al.*^40^ Similarly, the article by Rowland *et al.*^54^ was excluded due to overlap with the larger sample reported in Rowland *et al.*^43^ Data from a single study were reported across two articles^55, 56^ from which data extraction was combined. The clinical characteristics of the ^1^H-MRS samples are provided in Table 1. Data used to calculate effect sizes are available in Supplementary Table 1. The methodological characteristics are provided in Supplementary Table 2.

### Medial frontal cortex

**Figure 2:**
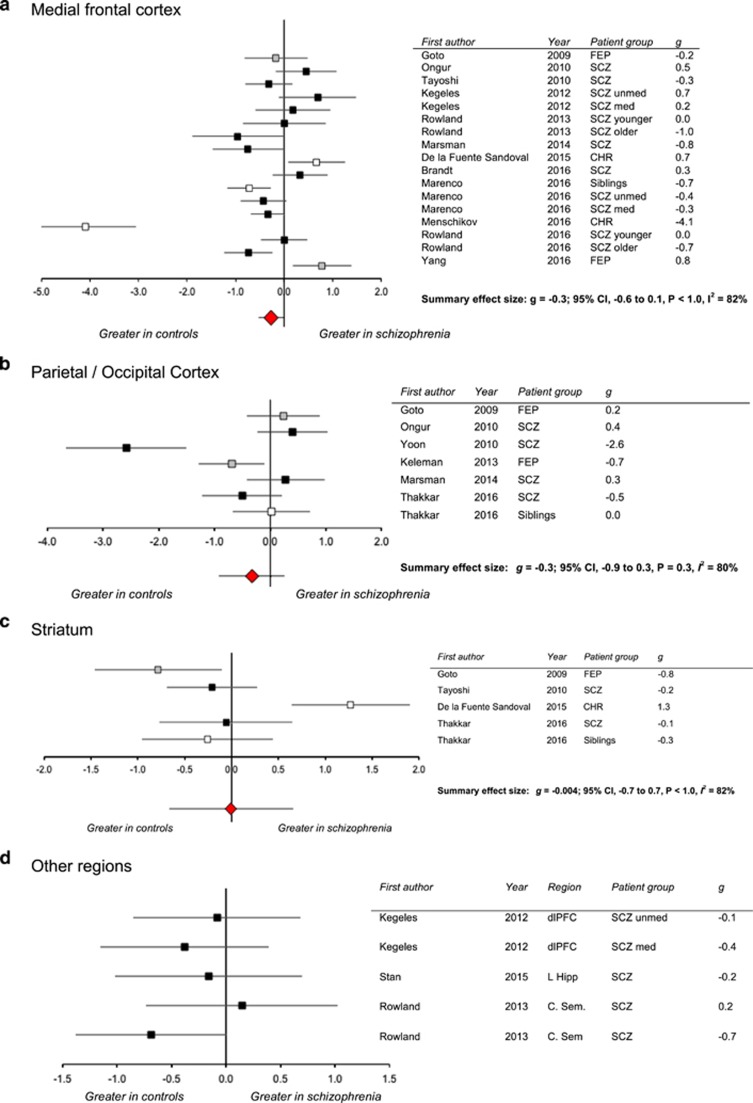
(**a**–**d**) Forest plots showing effect sizes (Hedge’s *g*) for ^1^H-MRS GABA studies in schizophrenia versus control. Error bars represent 95% confidence intervals. Black squares indicate data from clinical patient samples (FEP or SCZ) while white squares indicate data from CHR or sibling samples. CHR, clinical high risk; FEP, first episode psychosis; ^1^H-MRS, proton magnetic resonance spectroscopy; SCZ, schizophrenia or schizoaffective disorder; sibling, unaffected siblings of patients with schizophrenia; med, currently receiving antipsychotic medication; unmed, currently unmedicated with antipsychotics.

Twelve articles^38, 40, 41, 42, 43, 55, 57, 58, 59, 60, 61, 62^ involved 17 data sets for GABA+ in the mFC, providing data from a total of 409 patients and 495 controls. Meta-analysis returned a summary effect size of *g*=−0.3, which was nonsignificant (95% confidence interval: −0.6 to 0.1, *P*=0.1, Figure 2a). The *I*^2^ value was 84%, indicating a significant (*P*<0.001) and considerable heterogeneity across data sets.^50^ Visual inspection of the Forrest plot (Figure 2a) shows that one study^61^ was clearly an outlier, and that of the remaining studies, approximately half reported higher GABA+ levels in patients than controls, while the other half reported the opposite. The recalculated summary effect size after removal of the outlying data set was *g*=−0.1, which was also nonsignificant (95% confidence interval: −0.4 to 0.2, *P*=0.5, *I*^2^=69%). Sensitivity analyses did not return significant results on any iteration, and the Eggers test did not suggest publication bias.

Available data sets permitted a series of subgroup analyses, which involved studies that (i) only included patients with a first episode of psychosis or schizophrenia (14 data sets^38, 40, 42, 43, 55, 57, 58, 59, 60, 62^); (ii) explicitly excluded patents taking benzodiazepine or anticonvulsant medication (eight data sets^38, 41, 42, 43^); (iii) included patients of whom >90% were being treated with antipsychotic medication (12 data sets^38, 40, 42, 43, 55, 57, 58, 60^); (iv) excluded subjects who had taken antipsychotic medication (five data sets^38, 40, 41, 62^); or (v) included only 1H-MRS voxels in the medial prefrontal area of the medial frontal cortex (Figure 1; 10 data sets^40, 41, 42, 43, 57, 59, 60^). All of these subgroup analyses returned nonsignificant summary effect sizes and *I*^2^ values >50%. Meta-regression did not reveal any significant relationships between mFC GABA+ and age, illness duration, symptom severity, percentage grey matter in the voxel or publication date. There was a significant association with percentage of males in the sample (17 observations, *β*=−0.04; *t*=−2.5; *P*=0.03), but this was driven by outlying values from one study that included only male subjects.^61^ The effect was no longer significant when this study had been removed (*β*=−0.004; *t*=−0.4; *P*=0.7).

### Parietal/occipital cortex

Meta-analysis of GABA+ in the parietal/occipital cortex included seven observations across six articles,^39, 55, 57, 59, 63, 64^ providing data from a total of 139 patients and 111 controls. The summary effect size was nonsignificant (*g*=−0.3; 95% confidence interval: −0.9 to 0.3, *P*=0.3, *I*^2^=80% Figure 2b) with no indication of publication bias. Limiting the analysis to observations in first episode psychosis or schizophrenia (six observations^39, 55, 57, 59, 63, 64^) also returned nonsignificant summary effect sizes. There were insufficient data to investigate further subgroups. All meta-regression analyses were nonsignificant.

### Striatum

Five data sets reported GABA+ in the striatum across four articles.^39, 41, 55, 58^ The summary effect size was not significant (123 patients, 95 controls, *g*=−0.004; 95% confidence interval: −0.7 to 0.7, *P*<1.0, *I*^2^=82% Figure 2c), with no indication of publication bias. There were insufficient data for subgroup analyses and meta-regression returned nonsignificant findings.

### Other brain regions

One study^40^ examined GABA+ in the dorsolateral prefrontal cortex, one^42^ examined GABA in the centrum semiovale and one^65^ examined GABA+ in the left hippocampus (Table 1, Figure 2d). There were insufficient data for meta-analysis and no significant group differences in GABA+ were reported for these brain regions.

### GABA_A_/BZR availability

**Figure 3:**
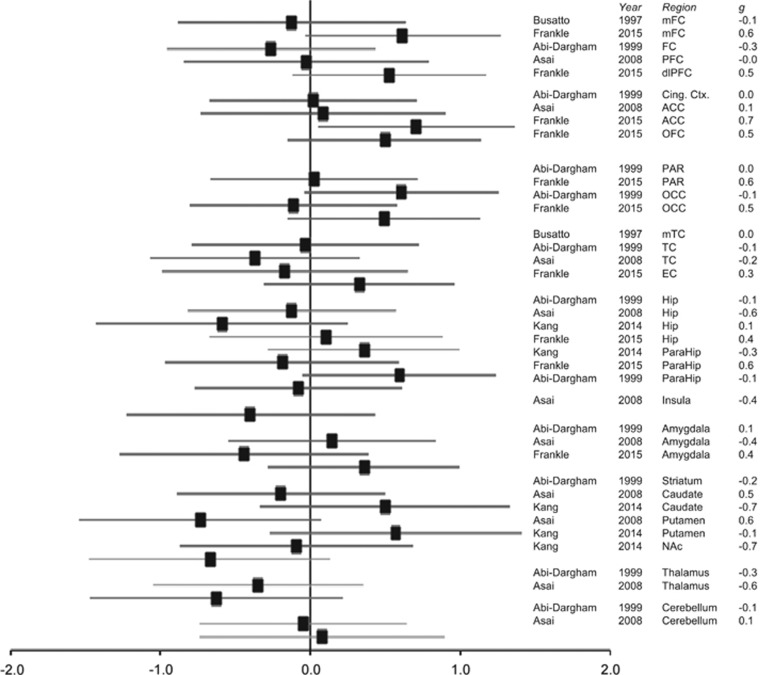
Illustration of effect sizes (Hedge’s *g*) for PET/SPECT studies of regional GABA_A_/BDZ receptor availability in schizophrenia versus control. Error bars represent 95% confidence intervals. ACC, anterior cingulate cortex; Cing. Ctx, cingulate cortex; dlPFC, dorsolateral prefrontal cortex; EC, entorhinal cortex; FC, frontal cortex; Hip, hippocampus; ^1^H-MRS, proton magnetic resonance spectroscopy; mFC, medial frontal cortex; mTC, medial temporal cortex; NAc, nucleus accumbens; OFC, orbitofrontal cortex; ParaHip, parahippocampus; PFC, prefrontal cortex; TC, temporal cortex. Studies reporting only voxel-wise analyses^70, 74^ are excluded from the figure.

Ten articles were initially identified, which reported GABA_A_/BZR availability in schizophrenia.^66, 67, 68, 69, 70, 71, 72, 73, 74, 75^ Of these, three were excluded: one because it was a conference abstract rather than a paper,^66^ one because it presented previously published data^69^ and one because it did not include a control group^68^ (Supplementary Figure 1). The clinical and methodological characteristics of the remaining seven articles^67, 70, 71, 72, 73, 74, 75^ are provided in Table 2 and Supplementary Table 3, respectively. There were not sufficient ROI data to permit meta-analyses in any brain region. None of the individual ROI studies detected any significant differences in regional GABA_A_/BDZ receptor availability between patients and controls (Figure 3).^67, 71, 72, 75^ Of the voxel-wise studies, one reported significantly lower GABA_A_/BZR availability in clinical high-risk subjects in the right caudate nucleus,^73^ one reported lower GABA_A_/BDZ receptor availability in the left precentral gyrus in schizophrenia,^70^ and one reported decreased GABA_A_/BDZ receptor availability in the subgenual cingulate cortex and left temporal pole, but increased GABA_A_/BDZ receptor availability in the right inferior occipital gyrus in schizophrenia.^74^

Frankle *et al.*^75^ compared antipsychotic-naive and antipsychotic-exposed schizophrenia, finding elevated baseline GABA_A_/BZR availability in the antipsychotic-naive group across all brain regions investigated. Lee *et al.*^74^ compared patients with schizophrenia currently taking aripiprazole or risperidone, and detected lower GABA_A_/BZR availability in the right medial, dorsolateral prefrontal, frontal polar and right premotor cortices in the aripiprazole group.

Three articles examined the relationship between GABA_A_/BZR availability and symptom severity.^70, 71, 75^ None of these found significant associations. One article reported inverse relationships between positive symptoms and receptor binding in the medial temporal lobe, and between negative symptoms and binding in the medial frontal region.^67^ Another article reported an inverse relationship between receptor binding in the prefrontal cortex and hippocampus and negative symptom severity.^72^

Frankle *et al.*^75^ also examined the change in [^11^C] flumazenil V_T_ following administration of the GABA transporter inhibitor tiagabine to increase GABA levels. This study detected no difference between the overall schizophrenia group compared with controls, but a smaller tiagabine-induced change in V_T_ (GABA increase) in antipsychotic-naive patients, but not in antipsychotic-exposed patients, compared with controls.^75^

## Discussion

The main finding of this article is that meta-analyses of ^1^H-MRS studies found no evidence for significantly altered GABA+ concentrations in patients with schizophrenia compared with healthy volunteers in the medial frontal cortex, parieto-occipital cortex or striatum. Analyses revealed a substantial level of heterogeneity across studies, which may relate to differences in patient samples and ^1^H-MRS methodological characteristics. Although there were insufficient studies of GABA_A_/BZR availability to perform meta-analysis, a systematic review of these studies found no consistent evidence for altered GABA_A_/BZR availability in schizophrenia.

To our knowledge, this is the first meta-analysis of ^1^H-MRS GABA studies in schizophrenia. Postmortem studies in schizophrenia find reductions in GAD67,^1, 2, 3^ which is responsible for the majority of basal GABA synthesis in the cortex.^6^ The ^1^H-MRS GABA signal may reflect the entire GABA content of the voxel (that is, intracellular and extracellular, and involved in metabolism or neurotransmission). Recent work argues that the ^1^H-MRS GABA signal predominantly relates to extracellular, extra-synaptic GABA providing tonic inhibitory tone, rather than GABA involved in phasic synaptic neurotransmission.^76, 77^ Theoretically, the ^1^H-MRS GABA signal should therefore be sensitive to GAD67 reduction. However, our meta-analysis of *in vivo* ^1^H-MRS GABA studies in schizophrenia found that, although in cortical regions the summary effect sizes were consistent with lower GABA levels, these effect sizes were small and nonsignificant.

An absence of large, detectable differences in GABA concentrations in schizophrenia *in vivo* could reflect normalization by compensatory mechanisms at the cellular or network level,^15^ and it is unknown whether GAD67 reduction in schizophrenia is primary, or secondary to other pathological mechanisms such as glutamatergic dysfunction.^78, 79^ Furthermore, one limitation of ^1^H-MRS is that it measures total GABA concentrations within a relatively large voxel (mean 30 ml in the studies included in this article), which is determined *a priori*, and cannot discriminate between GABA levels in different cell types. This limits the application of ^1^H-MRS in addressing the cell- and network- specific GABA abnormalities hypothesized to occur in schizophrenia.^15^

The ^1^H-MRS meta-analysis also reflects several limitations in the currently available literature. Sixteen studies contributed to the meta-analysis, but there were relatively few investigations in each brain region, with non-overlapping voxel placements (for example in the mFC), variability between clinical samples and ^1^H-MRS methodological approaches and high heterogeneity. Meta-analysis revealed substantial variability in the findings across studies. For example, there were approximately equal numbers of studies reporting increases of GABA+ in the medial frontal cortex in schizophrenia as there were studies reporting reductions (Figure 2a), and all meta-analyses were associated with significant and high levels of heterogeneity. This may reflect between-study differences in patient samples, methodological approaches or relate to inconsistency in ^1^H-MRS GABA measurement. Regional brain GABA levels in schizophrenia may vary with the stage of the disorder, as has been reported in some individual studies,^38, 40, 42, 43^ and appears to be evident for brain glutamate levels.^80^ Our analysis was limited in that there were too few studies to perform meta-analyses of all patient subgroups in all regions. However, exclusion of data sets from ‘at risk’ participants (and thus restricting the analysis to patients with schizophrenia) did not change our findings. Similarly, the findings in the mFC remained nonsignificant and heterogeneous when the analysis was limited to either antipsychotic unmedicated or treated patients, or restricted to the prefrontal part of the medial frontal region. Moreover, meta-regression found no effect of duration of illness, participant age or symptom severity on GABA effect sizes. Nevertheless, there are several other clinical and methodological variables that might contribute to heterogeneity, such as the duration of treatment, time off medication or substance use, which we were not able to investigate in this meta-analysis. It is also possible that more complex relationships exist between two or more study variables on the GABA effect size, for example the location of GABA dysfunction within the mFC may vary with age or illness stage.

Owing to limited data availability, our meta-analysis did not account for the several methodological differences between studies that may have impacted on data quality. Differences in field strength, voxel size and acquisition times will translate to large between-study differences in the signal to noise ratio, and it was not possible to evaluate spectral quality in 7 of the 16 included articles (see Supplementary Information). Only two recent studies included methodology to isolate the GABA signal from macromolecule contamination,^39, 43^ which is a key area for future methodological development. Therefore, while the meta-analyses did not indicate differences in regional GABA levels between patients and controls, this interpretation is limited pending publication of further individual studies. Future studies should directly compare different patient samples, maximize signal to noise ratio, address macromolecule contamination and include detailed and transparent reporting of spectral quality. On the basis of postmortem evidence, we suggest that key regions for investigation include the dorsolateral prefrontal cortex, anterior cingulate cortex and hippocampus.^3, 10, 17, 81, 82, 83^

Our systematic review of PET/SPECT studies examining GABA_A_/BZR availability also suggested an overall lack of evidence for differences in patients compared with controls, with no significant regional group differences in four out of seven studies.^67, 71, 72, 75^ However, the three voxel-wise studies all reported lower GABA_A_/BZR availability in patients compared with controls, but there was no consistency across studies in the regions where these differences were detected.^70, 73, 74^ All identified studies applied PET/SPECT radiotracers with affinity at the BZ site of the GABA_A_/BZR complex, and postmortem autoradiography studies of availability of BZ binding sites have also produced inconsistent results.^22, 23, 24, 25, 26, 27^ This contrasts with reports of increases in availability of the GABA binding site on the GABA_A_/BZR in schizophrenia postmortem,^16, 17, 18, 19, 20, 21, 22^ for which *in vivo* radiotracers are currently unavailable. A further consideration is that several PET/SPECT studies estimated regional GABA_A_/BZR availability relative to white matter,^67, 72, 73, 74^ which may be confounded by the presence of white matter abnormalities in schizophrenia (see ref. 84).

In contrast to ^1^H-MRS, GABA_A_/BZR PET imaging may be able to measure changes in synaptic GABA concentrations. Frankle *et al.*^75^ used this approach to examine the increase in GABA following administration of the presynaptic GABA reuptake inhibitor tiagabine. Although they found no difference between patients with schizophrenia and controls, when the analysis was restricted to the subgroup of patients that were antipsychotic-naive, the increase in GABA following tiagabine was significantly diminished. Tiagabine-induced increases in cortical GABA are not detectable using ^1^H-MRS,^85, 86^ which is consistent with the view that the GABA ^1^H-MRS signal principally reflects nonsynaptic GABA. Pharmacologically induced alterations in synaptic GABA may be more sensitively imaged with [^11^C]Ro15-4513 PET, because it is a GABA_A_/BZR inverse agonist with greater selectivity for intra-synaptic receptors.^87^ In the future, combination of this approach with ^1^H-MRS in the same subjects, and potentially during the same scanning session on combined PET-MR platforms, might investigate dysfunction of synaptic versus nonsynaptic GABA in schizophrenia.

Cluster analyses of postmortem data find that GABAergic deficits are not present in all schizophrenia patients, but characterize a patient subgroup of approximately 50% of the postmortem sample.^88, 89^ This postmortem ‘Low GABA Marker’ (LGM) phenotype^89^ does not readily relate to illness severity, psychoactive medication or substance use at the time of death.^88, 89^ If a LGM subgroup could similarly be identified using *in vivo* biomarkers, this might lead to a stratified approach to treatments that address GABAergic dysfunction. It is possible that the heterogeneity in ^1^H-MRS studies may also reflect GABAergic subgroups of patients, either within- or between-study samples, which are again not readily identifiable by clinical variables. However, unlike postmortem studies, GABA imaging studies did not show consistently higher variability in GABA measurements in the patient compared with the control group (Supplementary Table 2). Combination of GABA ^1^H-MRS or GABA PET/SPECT with electroencephalogram gamma-band oscillations in schizophrenia,^53, 75, 90^ which reflect on parvalbumin neuron activity,^91^ may help determine whether such GABAergic subgroups of patients are identifiable *in vivo*.

In conclusion, at present, the neuroimaging literature suggests that brain GABA function, as indexed by ^1^H-MRS GABA concentrations and GABA_A_/BZR BZ site availability, does not provide a consistent pattern of alteration in schizophrenia. However, the total number of studies completed in this field is still relatively small, and most studies to date have involved small patient samples (typically 15–30 patients), and varying data quality (see Supplementary Information for discussion). It remains unclear if the absence of overall differences reflects confounding effects of age, stage of illness, medications or other unknown factors. Further studies using larger and more homogeneous samples may therefore be useful, as would studies directly comparing specific patient subgroups. Advances in both ^1^H-MRS and PET methodologies may reveal specific aspects of GABA dysfunction *in vivo* in schizophrenia within the next few years.

## Figures and Tables

**Table 1:** ^1^H-MRS GABA data sets: clinical characteristics of the samples

*Region*	*First author* *(reference)*	*Year*	*Patient group*	g	*Sample size*		*SCZ* *%M*	*SCZ age* *mean*	*%AP*	*%BZ*	*PANSS*	*DOI*
					*C*	*SCZ*						
mFC	Goto^55, 56^	2009	FEP	−0.2	18	18	50	29	100	NR	68	0.5
	Öngür^57^	2010	SCZ	0.5	19	21	67	39	95	76	51	21
	Tayoshi^58^	2010	SCZ	−0.3	29	38	53	34	100	42	51	21
	Kegeles^40^	2012	SCZ unmed	0.7	11	16	69	32	0	19	71	7
	Kegeles^40^	2012	SCZ med	0.2	11	16	69	32	100	19	57	9
	Rowland^42^	2013	SCZ young	0.0	10	11	82	30	100	0	63	7.7
	Rowland^42^	2013	SCZ old	−1.0	10	10	70	51	100	0	57	25.5
	Marsman^59^	2014	SCZ	−0.8	19	13	76	28	100	35	53	6.5
	De la Fuente Sandoval^41^	2015	CHR	0.7	24	23	65	21	0	0	NR	—
	Brandt^60^	2016	SCZ	0.3	24	24	79	38	100	17	NR	NR
	Marenco^38^	2016	Siblings	−0.7	61.3	31	55	30	0	0	NR	—
	Marenco^38^	2016	SCZ unmed	−0.4	61.3	25	72	28	0	0	NR	6.0
	Marenco^38^	2016	SCZ med	−0.3	61.3	70	71	31	100	36	NR	9.5
	Menschikov^61^	2016	CHR	−4.1	26	21	100	NR	NR	NR	NR	—
	Rowland^43^	2016	SCZ young	0.0	40	29	69	26	93	0	NR	5.6
	Rowland^43^	2016	SCZ old	−0.7	37	31	61	48	90	0	NR	24
	Yang^62^	2016	FEP	0.8	23	22	41	26	0	NR	69	1.6
												
POC	Goto^55, 56^	2009	FEP	0.2	18	18	50	29	100	NR	68	0.5
	Öngür^57^	2010	SCZ	0.4	19	21	67	39	95	76	51	21
	Yoon^63^	2010	FEP/SCZ	−2.6	13	13	85	28	62	NR	73	NR
	Kelemen^64^	2013	FEP	−0.7	20	28	64	25	0	0	88	0.8
	Marsman^59^	2014	SCZ	0.3	19	15	76	28	100	35	53	6.5
	Thakkar^39^	2016	Sibling	0.0	12	23	53	31	0	0	NR	—
	Thakkar^39^	2016	SCZ	−0.5	12	21	71	36	100	24	49	14
												
Striatum	Goto^55, 56^	2009	FEP	−0.8	18	18	50	29	100	NR	68	0.5
	Tayoshi^58^	2010	SCZ	−0.2	29	38	53	34	100	42	51	21
	De la Fuente Sandoval^41^	2015	CHR	1.3	24	23	65	21	0	0	NR	—
	Thakkar^39^	2016	Sibling	−0.3	12	23	53	31	0	0	NR	—
	Thakkar^39^	2016	SCZ	−0.1	12	21	71	36	100	24	49	14
												
dlPFC	Kegeles^40^	2012	SCZ unmed	−0.1	11	16	69	32	0	19	71	7
dlPFC	Kegeles^40^	2012	SCZ med	−0.4	11	16	69	32	100	19	57	9
Hippocampus	Stan^65^	2015	SCZ	−0.2	16	18	78	42	61	28	NR	NR
CSO	Rowland^42^	2013	SCZ younger	0.2	10	11	82	30	100	0	63	7.7
CSO	Rowland^42^	2013	SCZ older	−0.7	10	10	70	51	1000	0	57	25.5

Abbreviations: %AP, percentage of SCZ group currently taking antipsychotic medication; %BZ, percentage of SCZ group currently taking benzodiazepine or anticonvulsant medication; C, control; CHR, clinical high risk; CSO, centrum semiovale; dlPFC, dorsolateral prefrontal cortex; DOI, mean duration of illness in years; FEP, first episode psychosis; *g*, Hedge’s *g* effect size; %M, percentage of male in the SCZ sample; mFC, medial frontal cortex area; NR, not reported; PANSS, Positive and Negative Syndrome Scale mean total symptom score; POC, parietal/occipital cortex; SCZ, schizophrenia or schizophreniform disorder; sibling, healthy siblings of patients with SCZ. Age is expressed in years (mean).

**Table 2:** PET/SPECT GABA_A_/BDZ receptor availability data sets: clinical characteristics of the samples

*First author*	*Year*	*Patient group*	*Sample size*		*SCZ* *%M*	*SCZ* *age* *mean*	*%AP*	*%BZ*	*PANSS*	*DOI*
			*C*	*SCZ*						
Busatto^67^	1997	SCZ	12	15	93	29	60	NR	NR	6.5
Verhoeff^70^	1999	SCZ	24	25	100	41	80	0	NR	NR
Abi-Dargham^71^	1999	SCZ	16	16	100	44	69	0	NR	NR
Asai^72^	2008	SCZ	11	12	55	33	0	0	90.4	NR
Lee^74^	2013	SCZ	18	17	47	29	100	0	60.5	4.1
Kang^73^	2014	CHR	15	11	66	19	18	0	NR	—
Frankle^75^	2015	SCZ	22	17	65	28	0	NR	83	NR

Abbreviations: %AP, percentage of SCZ group currently taking antipsychotic medication; %BZ, percentage of SCZ group currently taking benzodiazepine or anticonvulsant medication; C, control; CHR, clinical high risk; DOI, mean duration of illness in years; %M, percentage of male in SCZ sample; NR, not reported; PANSS, Positive and Negative Syndrome Scale mean total symptom score; SCZ, schizophrenia. Age is expressed in years, mean; regional effect sizes are provided in Figure 2.

## References

[bib1] Hashimoto T, Volk DW, Eggan SM, Mirnics K, Pierri JN, Sun Z et al. Gene expression deficits in a subclass of GABA neurons in the prefrontal cortex of subjects with schizophrenia. J Neurosci 2003; 23: 6315–6326. 12867516 10.1523/JNEUROSCI.23-15-06315.2003 PMC6740534

[bib2] Volk DW, Austin MC, Pierri JN, Sampson AR, Lewis DA. Decreased glutamic acid decarboxylase67 messenger RNA expression in a subset of prefrontal cortical gamma-aminobutyric acid neurons in subjects with schizophrenia. Arch Gen Psychiatry 2000; 57: 237–245. 10711910 10.1001/archpsyc.57.3.237

[bib3] Akbarian S, Kim JJ, Potkin SG, Hagman JO, Tafazzoli A, Bunney WEJr et al. Gene expression for glutamic acid decarboxylase is reduced without loss of neurons in prefrontal cortex of schizophrenics. Arch Gen Psychiatry 1995; 52: 258–266. 7702443 10.1001/archpsyc.1995.03950160008002

[bib4] Esclapez M, Tillakaratne NJ, Kaufman DL, Tobin AJ, Houser CR. Comparative localization of two forms of glutamic acid decarboxylase and their mRNAs in rat brain supports the concept of functional differences between the forms. J Neurosci 1994; 14(3 Pt 2): 1834–1855. 8126575 10.1523/JNEUROSCI.14-03-01834.1994 PMC6577546

[bib5] Battaglioli G, Liu H, Martin DL. Kinetic differences between the isoforms of glutamate decarboxylase: implications for the regulation of GABA synthesis. J Neurochem 2003; 86: 879–887. 12887686 10.1046/j.1471-4159.2003.01910.x

[bib6] Asada H, Kawamura Y, Maruyama K, Kume H, Ding RG, Kanbara N et al. Cleft palate and decreased brain gamma-aminobutyric acid in mice lacking the 67-kDa isoform of glutamic acid decarboxylase. Proc Natl Acad Sci USA 1997; 94: 6496–6499. 9177246 10.1073/pnas.94.12.6496 PMC21078

[bib7] Benes FM, Todtenkopf MS, Logiotatos P, Williams M. Glutamate decarboxylase(65)-immunoreactive terminals in cingulate and prefrontal cortices of schizophrenic and bipolar brain. J Chem Neuroanat 2000; 20: 259–269. 11207424 10.1016/s0891-0618(00)00105-8

[bib8] Glausier JR, Kimoto S, Fish KN, Lewis DA. Lower glutamic acid decarboxylase 65-kDa isoform messenger RNA and protein levels in the prefrontal cortex in schizoaffective disorder but not schizophrenia. Biol Psychiatry 2015; 77: 167–176. 24993056 10.1016/j.biopsych.2014.05.010 PMC4247819

[bib9] Dracheva S, Elhakem SL, McGurk SR, Davis KL, Haroutunian V. GAD67 and GAD65 mRNA and protein expression in cerebrocortical regions of elderly patients with schizophrenia. J Neurosci Res 2004; 76: 581–592. 15114630 10.1002/jnr.20122

[bib10] Hashimoto T, Bazmi HH, Mirnics K, Wu Q, Sampson AR, Lewis DA. Conserved regional patterns of GABA-related transcript expression in the neocortex of subjects with schizophrenia. Am J Psychiatry 2008; 165: 479–489. 18281411 10.1176/appi.ajp.2007.07081223 PMC2894608

[bib11] Tian N, Petersen C, Kash S, Baekkeskov S, Copenhagen D, Nicoll R. The role of the synthetic enzyme GAD65 in the control of neuronal gamma-aminobutyric acid release. Proc Natl Acad Sci USA 1999; 96: 12911–12916. 10536022 10.1073/pnas.96.22.12911 PMC23160

[bib12] Patel AB, de Graaf RA, Martin DL, Battaglioli G, Behar KL. Evidence that GAD65 mediates increased GABA synthesis during intense neuronal activity *in vivo*. J Neurochem 2006; 97: 385–396. 16539672 10.1111/j.1471-4159.2006.03741.x

[bib13] Tse MT, Piantadosi PT, Floresco SB. Prefrontal cortical gamma-aminobutyric acid transmission and cognitive function: drawing links to schizophrenia from preclinical research. Biol Psychiatry 2015; 77: 929–939. 25442792 10.1016/j.biopsych.2014.09.007

[bib14] Lisman JE, Coyle JT, Green RW, Javitt DC, Benes FM, Heckers S et al. Circuit-based framework for understanding neurotransmitter and risk gene interactions in schizophrenia. Trends Neurosci 2008; 31: 234–242. 18395805 10.1016/j.tins.2008.02.005 PMC2680493

[bib15] Lewis DA, Curley AA, Glausier JR, Volk DW. Cortical parvalbumin interneurons and cognitive dysfunction in schizophrenia. Trends Neurosci 2012; 35: 57–67. 22154068 10.1016/j.tins.2011.10.004 PMC3253230

[bib16] Hanada S, Mita T, Nishino N, Tanaka C. [3H]muscimol binding sites increased in autopsied brains of chronic schizophrenics. Life Sci 1987; 40: 259–266. 3025545 10.1016/0024-3205(87)90341-9

[bib17] Benes FM, Vincent SL, Alsterberg G, Bird ED, SanGiovanni JP. Increased GABAA receptor binding in superficial layers of cingulate cortex in schizophrenics. J Neurosci 1992; 12: 924–929. 1372045 10.1523/JNEUROSCI.12-03-00924.1992 PMC6576044

[bib18] Benes FM, Vincent SL, Marie A, Khan Y. Up-regulation of GABAA receptor binding on neurons of the prefrontal cortex in schizophrenic subjects. Neuroscience 1996; 75: 1021–1031. 8938738 10.1016/0306-4522(96)00328-4

[bib19] Dean B, Hussain T, Hayes W, Scarr E, Kitsoulis S, Hill C et al. Changes in serotonin2A and GABA(A) receptors in schizophrenia: studies on the human dorsolateral prefrontal cortex. J Neurochem 1999; 72: 1593–1599. 10098866 10.1046/j.1471-4159.1999.721593.x

[bib20] Deng C, Huang XF. Increased density of GABAA receptors in the superior temporal gyrus in schizophrenia. Exp Brain Res 2006; 168: 587–590. 16362364 10.1007/s00221-005-0290-9

[bib21] Newell KA, Zavitsanou K, Jew SK, Huang XF. Alterations of muscarinic and GABA receptor binding in the posterior cingulate cortex in schizophrenia. Prog Neuropsychopharmacol Biol Psychiatry 2007; 31: 225–233. 16901598 10.1016/j.pnpbp.2006.07.004

[bib22] Verdurand M, Fillman SG, Weickert CS, Zavitsanou K. Increases in [3H]muscimol and [3H]flumazenil binding in the dorsolateral prefrontal cortex in schizophrenia are linked to alpha4 and gamma2S mRNA levels respectively. PLoS ONE 2013; 8: e52724. 23320076 10.1371/journal.pone.0052724 PMC3540049

[bib23] Squires RF, Lajtha A, Saederup E, Palkovits M. Reduced [3H]flunitrazepam binding in cingulate cortex and hippocampus of postmortem schizophrenic brains: is selective loss of glutamatergic neurons associated with major psychoses? Neurochem Res 1993; 18: 219–223. 8097289 10.1007/BF01474687

[bib24] Kiuchi Y, Kobayashi T, Takeuchi J, Shimizu H, Ogata H, Toru M. Benzodiazepine receptors increase in post-mortem brain of chronic schizophrenics. Eur Arch Psychiatry Neurol Sci 1989; 239: 71–78. 2553417 10.1007/BF01759578

[bib25] Reynolds GP, Stroud D. Hippocampal benzodiazepine receptors in schizophrenia. J Neural Transm Gen Sect 1993; 93: 151–155. 8217052 10.1007/BF01245344

[bib26] Pandey GN, Conley RR, Pandey SC, Goel S, Roberts RC, Tamminga CA et al. Benzodiazepine receptors in the post-mortem brain of suicide victims and schizophrenic subjects. Psychiatry Res 1997; 71: 137–149. 9271787 10.1016/s0165-1781(97)00060-7

[bib27] Benes FM, Wickramasinghe R, Vincent SL, Khan Y, Todtenkopf M. Uncoupling of GABA(A) and benzodiazepine receptor binding activity in the hippocampal formation of schizophrenic brain. Brain Res 1997; 755: 121–129. 9163547 10.1016/s0006-8993(97)00113-3

[bib28] Glausier JR, Lewis A. Selective pyramidal cell reduction of GABA(A) receptor alpha1 subunit messenger RNA expression in schizophrenia. Neuropsychopharmacology 2011; 36: 2103–2110. 21677653 10.1038/npp.2011.102 PMC3158308

[bib29] Beneyto M, Abbott A, Hashimoto T, Lewis DA. Lamina-specific alterations in cortical GABA(A) receptor subunit expression in schizophrenia. Cereb Cortex 2011; 21: 999–1011. 20843900 10.1093/cercor/bhq169 PMC3077427

[bib30] Volk DW, Pierri JN, Fritschy JM, Auh S, Sampson AR, Lewis DA. Reciprocal alterations in pre- and postsynaptic inhibitory markers at chandelier cell inputs to pyramidal neurons in schizophrenia. Cereb Cortex 2002; 12: 1063–1070. 12217970 10.1093/cercor/12.10.1063

[bib31] Impagnatiello F, Guidotti AR, Pesold C, Dwivedi Y, Caruncho H, Pisu MG et al. A decrease of reelin expression as a putative vulnerability factor in schizophrenia. Proc Natl Acad Sci USA 1998; 95: 15718–15723. 9861036 10.1073/pnas.95.26.15718 PMC28110

[bib32] Akbarian S, Huntsman MM, Kim JJ, Tafazzoli A, Potkin SG, Bunney WEJr et al. GABAA receptor subunit gene expression in human prefrontal cortex: comparison of schizophrenics and controls. Cereb Cortex 1995; 5: 550–560. 8590827 10.1093/cercor/5.6.550

[bib33] Frankle WG, Cho RY, Narendran R, Mason NS, Vora S, Litschge M et al. Tiagabine increases [11C]flumazenil binding in cortical brain regions in healthy control subjects. Neuropsychopharmacology 2009; 34: 624–633. 18615011 10.1038/npp.2008.104 PMC2754778

[bib34] Lingford-Hughes A, Hume SP, Feeney A, Hirani E, Osman S, Cunningham VJ et al. Imaging the GABA-benzodiazepine receptor subtype containing the alpha5-subunit *in vivo* with [11C]Ro15 4513 positron emission tomography. J Cereb Blood Flow Metab 2002; 22: 878–889. 12142573 10.1097/00004647-200207000-00013

[bib35] Gill KM, Lodge DJ, Cook JM, Aras S, Grace AA. A novel alpha5GABA(A)R-positive allosteric modulator reverses hyperactivation of the dopamine system in the MAM model of schizophrenia. Neuropsychopharmacology 2011; 36: 1903–1911. 21562483 10.1038/npp.2011.76 PMC3154109

[bib36] Du Y, Grace AA. Loss of parvalbumin in the hippocampus of MAM schizophrenia model rats is attenuated by peripubertal diazepam. Int J Neuropsychopharmacol 2016; 19: pyw065. 27432008 10.1093/ijnp/pyw065 PMC5137280

[bib37] Marin O. Interneuron dysfunction in psychiatric disorders. Nat Rev Neurosci 2012; 13: 107–120. 22251963 10.1038/nrn3155

[bib38] Marenco S, Meyer C, Kuo S, van der Veen JW, Shen J, DeJong K et al. Prefrontal GABA levels measured with magnetic resonance spectroscopy in patients with psychosis and unaffected siblings. Am J Psychiatry 2016; 173: 527–534. 26806873 10.1176/appi.ajp.2015.15020190

[bib39] Thakkar KN, Rosler L, Wijnen JP, Boer VO, Klomp DW, Cahn W et al. 7T proton magnetic resonance spectroscopy of gamma-aminobutyric acid, glutamate, and glutamine reveals altered concentrations in patients with schizophrenia and healthy siblings. Biol Psychiatry 2016; 81: 525–535. 27316853 10.1016/j.biopsych.2016.04.007

[bib40] Kegeles LS, Mao X, Stanford AD, Girgis R, Ojeil N, Xu X et al. Elevated prefrontal cortex gamma-aminobutyric acid and glutamate-glutamine levels in schizophrenia measured *in vivo* with proton magnetic resonance spectroscopy. Arch Gen Psychiatry 2012; 69: 449–459. 22213769 10.1001/archgenpsychiatry.2011.1519

[bib41] de la Fuente-Sandoval C, Reyes-Madrigal F, Mao X, Leon-Ortiz P, Rodriguez-Mayoral O, Solis-Vivanco R et al. Cortico-striatal GABAergic and glutamatergic dysregulations in subjects at ultra-high risk for psychosis investigated with proton magnetic resonance spectroscopy. Int J Neuropsychopharmacol 2015; 19: pyv105. 26364273 10.1093/ijnp/pyv105 PMC4815472

[bib42] Rowland LM, Kontson K, West J, Edden RA, Zhu H, Wijtenburg SA et al. *In vivo* measurements of glutamate, GABA, and NAAG in schizophrenia. Schizophr Bull 2013; 39: 1096–1104. 23081992 10.1093/schbul/sbs092 PMC3756774

[bib43] Rowland LM, Krause BW, Wijtenburg SA, McMahon RP, Chiappelli J, Nugent KL et al. Medial frontal GABA is lower in older schizophrenia: a MEGA-PRESS with macromolecule suppression study. Mol Psychiatry 2016; 21: 198–204. 25824298 10.1038/mp.2015.34 PMC4591074

[bib44] Epperson CN, Haga K, Mason GF, Sellers E, Gueorguieva R, Zhang W et al. Cortical gamma-aminobutyric acid levels across the menstrual cycle in healthy women and those with premenstrual dysphoric disorder: a proton magnetic resonance spectroscopy study. Arch Gen Psychiatry 2002; 59: 851–858. 12215085 10.1001/archpsyc.59.9.851

[bib45] Moher D, Liberati A, Tetzlaff J, Altman DG, , PRISMA Group. Preferred reporting items for systematic reviews and meta-analyses: the PRISMA statement. J Clin Epidemiol 2009; 62: 1006–1012. 19631508 10.1016/j.jclinepi.2009.06.005

[bib46] Papadopoulos V, Baraldi M, Guilarte TR, Knudsen TB, Lacapere JJ, Lindemann P et al. Translocator protein (18kDa): new nomenclature for the peripheral-type benzodiazepine receptor based on its structure and molecular function. Trends Pharmacol Sci 2006; 27: 402–409. 16822554 10.1016/j.tips.2006.06.005

[bib47] Ioannidis J, Lau J. Evolution of treatment effects over time: empirical insight from recursive cumulative metaanalyses. Proc Natl Acad Sci USA 2001; 98: 831–836. 11158556 10.1073/pnas.021529998 PMC14669

[bib48] Hedges LV, Olkin I. Statistical Methods for Meta-Analysis. Academic Press: Orlando, FL, USA, 1985, xxii, 369p.

[bib49] DerSimonian R, Laird N. Meta-analysis in clinical trials. Control Clin Trials 1986; 7: 177–188. 3802833 10.1016/0197-2456(86)90046-2

[bib50] Higgins JP, Thompson SG, Deeks JJ, Altman DG. Measuring inconsistency in meta-analyses. BMJ 2003; 327: 557–560. 12958120 10.1136/bmj.327.7414.557 PMC192859

[bib51] Egger M, Davey Smith G, Schneider M, Minder C. Bias in meta-analysis detected by a simple, graphical test. BMJ 1997; 315: 629–634. 9310563 10.1136/bmj.315.7109.629 PMC2127453

[bib52] Leucht S, Rothe P, Davis JM, Engel RR. Equipercentile linking of the BPRS and the PANSS. Eur Neuropsychopharmacol 2013; 23: 956–959. 23433639 10.1016/j.euroneuro.2012.11.004

[bib53] Chen CM, Stanford AD, Mao X, Abi-Dargham A, Shungu DC, Lisanby SH et al. GABA level, gamma oscillation, and working memory performance in schizophrenia. Neuroimage Clin 2014; 4: 531–539. 24749063 10.1016/j.nicl.2014.03.007 PMC3989525

[bib54] Rowland LM, Summerfelt A, Wijtenburg SA, Du X, Chiappelli JJ, Krishna N et al. Frontal glutamate and gamma-aminobutyric acid levels and their associations with mismatch negativity and digit sequencing task performance in schizophrenia. JAMA Psychiatry 2016; 73: 166–174. 26720179 10.1001/jamapsychiatry.2015.2680 PMC4740214

[bib55] Goto N, Yoshimura R, Moriya J, Kakeda S, Ueda N, Ikenouchi-Sugita A et al. Reduction of brain gamma-aminobutyric acid (GABA) concentrations in early-stage schizophrenia patients: 3T Proton MRS study. Schizophr Res 2009; 112: 192–193. 19464152 10.1016/j.schres.2009.04.026

[bib56] Goto N, Yoshimura R, Kakeda S, Moriya J, Hori H, Hayashi K et al. No alterations of brain GABA after 6 months of treatment with atypical antipsychotic drugs in early-stage first-episode schizophrenia. Prog Neuropsychopharmacol Biol Psychiatry 2010; 34: 1480–1483. 20727934 10.1016/j.pnpbp.2010.08.007

[bib57] Ongur D, Prescot AP, McCarthy J, Cohen BM, Renshaw PF. Elevated gamma-aminobutyric acid levels in chronic schizophrenia. Biol Psychiatry 2010; 68: 667–670. 20598290 10.1016/j.biopsych.2010.05.016 PMC2942977

[bib58] Tayoshi S, Nakataki M, Sumitani S, Taniguchi K, Shibuya-Tayoshi S, Numata S et al. GABA concentration in schizophrenia patients and the effects of antipsychotic medication: a proton magnetic resonance spectroscopy study. Schizophr Res 2010; 117: 83–91. 20022731 10.1016/j.schres.2009.11.011

[bib59] Marsman A, Mandl RC, Klomp DW, Bohlken MM, Boer VO, Andreychenko A et al. GABA and glutamate in schizophrenia: A 7 T (1)H-MRS study. Neuroimage Clin 2014; 6: 398–407. 25379453 10.1016/j.nicl.2014.10.005 PMC4218940

[bib60] Brandt AS, Unschuld PG, Pradhan S, Lim IA, Churchill G, Harris AD et al. Age-related changes in anterior cingulate cortex glutamate in schizophrenia: A (1)H MRS Study at 7 Tesla. Schizophr Res 2016; 172: 101–105. 26925800 10.1016/j.schres.2016.02.017 PMC4821673

[bib61] Menschikov PE, Semenova NA, Ublinskiy MV, Akhadov TA, Keshishyan RA, Lebedeva IS et al. (1)H-MRS and MEGA-PRESS pulse sequence in the study of balance of inhibitory and excitatory neurotransmitters in the human brain of ultra-high risk of schizophrenia patients. Doklady Biochem Biophys 2016; 468: 168–172. 10.1134/S1607672916030029 27417711

[bib62] Yang Z, Zhu Y, Song Z, Mei L, Zhang J, Chen T et al. Comparison of the density of gamma-aminobutyric acid in the ventromedial prefrontal cortex of patients with first-episode psychosis and healthy controls. Shanghai Archives of psychiatry 2015; 27: 341–347. 27199525 10.11919/j.issn.1002-0829.215130 PMC4858505

[bib63] Yoon JH, Maddock RJ, Rokem A, Silver MA, Minzenberg MJ, Ragland JD et al. GABA concentration is reduced in visual cortex in schizophrenia and correlates with orientation-specific surround suppression. JNeurosci 2010; 30: 3777–3781. 20220012 10.1523/JNEUROSCI.6158-09.2010 PMC2846788

[bib64] Kelemen O, Kiss I, Benedek G, Keri S. Perceptual and cognitive effects of antipsychotics in first-episode schizophrenia: the potential impact of GABA concentration in the visual cortex. Prog Neuropsychopharmacol Biol Psychiatry 2013; 47: 13–19. 23954737 10.1016/j.pnpbp.2013.07.024

[bib65] Stan AD, Ghose S, Zhao C, Hulsey K, Mihalakos P, Yanagi M et al. Magnetic resonance spectroscopy and tissue protein concentrations together suggest lower glutamate signaling in dentate gyrus in schizophrenia. Mol Psychiatry 2015; 20: 433–439. 24912493 10.1038/mp.2014.54

[bib66] Lawrie S, Ebmeier KP, Verhoeff NP, van Royen EA, Johnstone EC, Goodwin GM. Benzodiazepie GABA-A receptor binding in schizophrenia - a study with single photon emission tomography and 123I-iomazenil. Schizophr Res 1996; 18: XIID4.

[bib67] Busatto GF, Pilowsky LS, Costa DC, Ell PJ, David AS, Lucey JV et al. Correlation between reduced *in vivo* benzodiazepine receptor binding and severity of psychotic symptoms in schizophrenia. Am J Psychiatry 1997; 154: 56–63. 8988959 10.1176/ajp.154.1.56

[bib68] Schroder J, Bubeck B, Demisch S, Sauer H. Benzodiazepine receptor distribution and diazepam binding in schizophrenia: an exploratory study. Psychiatry Res 1997; 68: 125–131. 9104759 10.1016/s0925-4927(96)02843-0

[bib69] Ball S, Busatto GF, David AS, Jones SH, Hemsley DR, Pilowsky LS et al. Cognitive functioning and GABAA/benzodiazepine receptor binding in schizophrenia: a 123I-iomazenil SPET study. Biol Psychiatry 1998; 43: 107–117. 9474443 10.1016/s0006-3223(97)00300-4

[bib70] Verhoeff NP, Soares JC, D'Souza CD, Gil R, Degen K, Abi-Dargham A et al. [123I]Iomazenil SPECT benzodiazepine receptor imaging in schizophrenia. Psychiatry Res 1999; 91: 163–173. 10641580 10.1016/s0925-4927(99)00027-x

[bib71] Abi-Dargham A, Laruelle M, Krystal J, D'Souza C, Zoghbi S, Baldwin RM et al. No evidence of altered *in vivo* benzodiazepine receptor binding in schizophrenia. Neuropsychopharmacology 1999; 20: 650–661. 10327433 10.1016/S0893-133X(98)00107-9

[bib72] Asai Y, Takano A, Ito H, Okubo Y, Matsuura M, Otsuka A et al. GABAA/Benzodiazepine receptor binding in patients with schizophrenia using [11C]Ro15-4513, a radioligand with relatively high affinity for alpha5 subunit. Schizophr Res 2008; 99: 1–3. 18042347 10.1016/j.schres.2007.10.014

[bib73] Kang JI, Park HJ, Kim SJ, Kim KR, Lee SY, Lee E et al. Reduced binding potential of GABA-A/benzodiazepine receptors in individuals at ultra-high risk for psychosis: an [18F]-fluoroflumazenil positron emission tomography study. Schizophr Bull 2014; 40: 548–557. 23588475 10.1093/schbul/sbt052 PMC3984508

[bib74] Lee JS, Lee JD, Park HJ, Oh MK, Chun JW, Kim SJ et al. Is the GABA system related to the social competence improvement effect of aripiprazole? An (18)F-Fluoroflumazenil PET study. Psychiatry Investig 2013; 10: 75–80. 10.4306/pi.2013.10.1.75 PMC3590434 23482902

[bib75] Frankle WG, Cho RY, Prasad KM, Mason NS, Paris J, Himes ML et al. *In vivo* measurement of GABA transmission in healthy subjects and schizophrenia patients. Am J Psychiatry 2015; 172: 1148–1159. 26133962 10.1176/appi.ajp.2015.14081031 PMC5070491

[bib76] Stagg CJ. Magnetic resonance spectroscopy as a tool to study the role of GABA in motor-cortical plasticity. Neuroimage 2014; 86: 19–27. 23333699 10.1016/j.neuroimage.2013.01.009

[bib77] Dyke K, Pepes SE, Chen C, Kim S, Sigurdsson HP, Draper A et al. Comparing GABA-dependent physiological measures of inhibition with proton magnetic resonance spectroscopy measurement of GABA using ultra-high-field MRI. Neuroimage 2017; 152: 360–370. 28284797 10.1016/j.neuroimage.2017.03.011 PMC5440178

[bib78] Belforte JE, Zsiros V, Sklar ER, Jiang Z, Yu G, Li Y et al. Postnatal NMDA receptor ablation in corticolimbic interneurons confers schizophrenia-like phenotypes. Nat Neurosci 2010; 13: 76–83. 19915563 10.1038/nn.2447 PMC2797836

[bib79] Turner CP, DeBenedetto D, Ware E, Stowe R, Lee A, Swanson J et al. Postnatal exposure to MK801 induces selective changes in GAD67 or parvalbumin. Exp Brain Res 2010; 201: 479–488. 19885653 10.1007/s00221-009-2059-z

[bib80] Merritt K, Egerton A, Kempton MJ, Taylor MJ, McGuire PK. Nature of glutamate alterations in schizophrenia: a meta-analysis of proton magnetic resonance spectroscopy studies. JAMA Psychiatry 2016; 73: 665–674. 27304221 10.1001/jamapsychiatry.2016.0442

[bib81] Maldonado-Aviles JG, Curley AA, Hashimoto T, Morrow AL, Ramsey AJ, O'Donnell P et al. Altered markers of tonic inhibition in the dorsolateral prefrontal cortex of subjects with schizophrenia. Am J Psychiatry 2009; 166: 450–459. 19289452 10.1176/appi.ajp.2008.08101484 PMC2887737

[bib82] Benes FM, Lim B, Matzilevich D, Walsh JP, Subburaju S, Minns M. Regulation of the GABA cell phenotype in hippocampus of schizophrenics and bipolars. Proc Natl Acad Sci USA 2007; 104: 10164–10169. 17553960 10.1073/pnas.0703806104 PMC1888575

[bib83] Knable MB, Barci BM, Webster MJ, Meador-Woodruff J, Torrey EF, , Stanley Neuropathology Consortium. Molecular abnormalities of the hippocampus in severe psychiatric illness: postmortem findings from the Stanley Neuropathology Consortium. Mol Psychiatry 2004; 9: 609–620, 544. 14708030 10.1038/sj.mp.4001471

[bib84] Walterfang M, Velakoulis D, Whitford TJ, Pantelis C. Understanding aberrant white matter development in schizophrenia: an avenue for therapy? Expert Rev Neurother 2011; 11: 971–987. 21721915 10.1586/ern.11.76

[bib85] Myers JF, Evans CJ, Kalk NJ, Edden RA, Lingford-Hughes AR. Measurement of GABA using J-difference edited 1H-MRS following modulation of synaptic GABA concentration with tiagabine. Synapse 2014; 68: 355–362. 24756906 10.1002/syn.21747

[bib86] Waschkies CF, Bruns A, Muller S, Kapps M, Borroni E, von Kienlin M et al. Neuropharmacological and neurobiological relevance of *in vivo* (1)H-MRS of GABA and glutamate for preclinical drug discovery in mental disorders. Neuropsychopharmacology 2014; 39: 2331–2339. 24694923 10.1038/npp.2014.79 PMC4138741

[bib87] Stokes PR, Myers JF, Kalk NJ, Watson BJ, Erritzoe D, Wilson SJ et al. Acute increases in synaptic GABA detectable in the living human brain: a [(1)(1)C]Ro15-4513 PET study. Neuroimage 2014; 99: 158–165. 24844747 10.1016/j.neuroimage.2014.05.035

[bib88] Volk DW, Matsubara T, Li S, Sengupta EJ, Georgiev D, Minabe Y et al. Deficits in transcriptional regulators of cortical parvalbumin neurons in schizophrenia. Am J Psychiatry 2012; 169: 1082–1091. 22983435 10.1176/appi.ajp.2012.12030305 PMC3513625

[bib89] Volk DW, Sampson AR, Zhang Y, Edelson JR, Lewis DA. Cortical GABA markers identify a molecular subtype of psychotic and bipolar disorders. Psychol Med 2016; 46: 2501–2512. 27328999 10.1017/S0033291716001446 PMC5584051

[bib90] Rowland LM, Edden RA, Kontson K, Zhu H, Barker PB, Hong LE. GABA predicts inhibition of frequency-specific oscillations in schizophrenia. J Neuropsychiatry Clin Neurosci 2013; 25: 83–87. 23487198 10.1176/appi.neuropsych.11120368 PMC3640485

[bib91] Sohal VS, Zhang F, Yizhar O, Deisseroth K. Parvalbumin neurons and gamma rhythms enhance cortical circuit performance. Nature 2009; 459: 698–702. 19396159 10.1038/nature07991 PMC3969859

