# Supplementary material for: Neuroimaging studies of GABA in schizophrenia: a systematic review with meta-analysis
Source: Transl Psychiatry. 2017 Jun 6;7(6):e1147–. doi: 10.1038/tp.2017.124 (PMC5537645; doi:10.1038/tp.2017.124)
Supplement: Supplementary Information [file tp2017124x1.docx]

**Supplementary Information**

Neuroimaging studies of GABA in Schizophrenia: A Systematic Review and Meta-Analysis Egerton, Modinos, Ferrera and McGuire.

**Supplementary Figure 1.** PRISMA diagram illustrating the literature search and article inclusion process.

| **Region** | **First**  **Author** | **Year** | **Patient group** | **Sample size** | | **C** | | **SCZ** | | ***g*** |
| --- | --- | --- | --- | --- | --- | --- | --- | --- | --- | --- |
|  |  |  |  | **C** | **SCZ** | **Mean** | **SD** | **Mean** | **SD** |  |
| mFC | Goto[^1^](#_ENREF_1)^,^ [^2^](#_ENREF_2) | 2009 | FEP | 18 | 18 | 1.30 | 1.16 | 1.12 | 0.88 | -0.2 |
|  | Öngür^[3](#_ENREF_3)^ | 2010 | SCZ | 19 | 21 | 0.10 | 0.03 | 0.12 | 0.03 | 0.5 |
|  | Tayoshi^[4](#_ENREF_4)^ | 2010 | SCZ | 29 | 38 | 1.52 | 0.54 | 1.36 | 0.45 | -0.3 |
|  | Kegeles^[5](#_ENREF_5)^ | 2012 | SCZ unmed | 11 | 16 | 2.02 x 10^-3^ | 0.30 x 10^-3^ | 2.69 x 10^-3^ | 1.19 x 10^-3^ | 0.7 |
|  | Kegeles^[5](#_ENREF_5)^ | 2012 | SCZ med | 11 | 16 | 2.02 x 10^-3^ | 0.30 x 10^-3^ | 2.14 x 10^-3^ | 0.78 x 10^-3^ | 0.2 |
|  | Rowland[^6^](#_ENREF_6) | 2013 | SCZ young | 10 | 11 | 1.30 | 0.30 | 1.30 | 0.20 | 0.0 |
|  | Rowland[^6^](#_ENREF_6) | 2013 | SCZ old | 10 | 10 | 1.20 | 0.20 | 1.00 | 0.20 | -1.0 |
|  | Marsman^[7](#_ENREF_7)^ | 2014 | SCZ | 19 | 13 | 1.52 | 0.54 | 1.36 | 0.45 | -0.8 |
|  | de la Fuente Sandoval[^8^](#_ENREF_8) | 2015 | CHR | 24 | 23 | 1.91 x 10^-3^ | 0.30 x 10^-3^ | 2.10 x 10^-3^ | 0.33 x 10^-3^ | 0.7 |
|  | Brandt[^9^](#_ENREF_9) | 2016 | SCZ | 24 | 24 | 2.24 | 0.31 | 2.36 | 0.41 | 0.3 |
|  | Marenco^[10](#_ENREF_10)^ | 2016 | Sibling | 61.3 | 31 | 9.73 | 0.91 | 9.07 | 0.90 | -0.7 |
|  | Marenco^[10](#_ENREF_10)^ | 2016 | SCZ unmed | 61.3 | 25 | 9.73 | 0.91 | 9.34 | 0.91 | -0.4 |
|  | Marenco^[10](#_ENREF_10)^ | 2016 | SCZ med | 61.3 | 70 | 9.73 | 0.91 | 9.42 | 0.92 | -0.3 |
|  | Menschikov^[11](#_ENREF_11)^ | 2016 | CHR | 26 | 21 | 0.20 | 0.01 | 0.17 | 0.01 | -4.1 |
|  | Rowland[^12^](#_ENREF_12) | 2016 | SCZ young | 40 | 29 | 0.97 | 0.15 | 0.97 | 0.11 | 0.0 |
|  | Rowland[^12^](#_ENREF_12) | 2016 | SCZ old | 37 | 31 | 0.89 | 0.17 | 0.77 | 0.15 | -0.7 |
|  | Yang[^13^](#_ENREF_13) | 2016 | FEP | 23 | 22 | 1.93 | 0.32 | 2.28 | 0.54 | 0.8 |
| POC | Goto[^1^](#_ENREF_1)^,^ [^2^](#_ENREF_2) | 2009 | FEP | 18 | 18 | 0.75 | 0.44 | 0.85 | 0.39 | 0.2 |
|  | Öngür^[3](#_ENREF_3)^ | 2010 | SCZ | 19 | 21 | 6.4 x 10^-2^ | 1.6 x 10^-2^ | 7.1 x 10^-2^ | 1.8 x 10^-2^ | 0.4 |
|  | Yoon[^14^](#_ENREF_14) | 2010 | FEP/SCZ | 13 | 13 | 1.05 x 10^-1^ | 0.05 x 10^-1^ | 0.94 x 10^-1^ | 0.03 x 10^-1^ | -2.6 |
|  | Kelemen^[15](#_ENREF_15)^ | 2013 | FEP | 20 | 28 | 0.11 | 0.03 | 0.08 | 0.05 | -0.7 |
|  | Marsman^[7](#_ENREF_7)^ | 2014 | SCZ | 19 | 15 | 0.14 | 0.03 | 0.15 | 0.04 | 0.3 |
|  | Thakkar^[16](#_ENREF_16)^ | 2016 | Sibling | 12 | 23 | 2.55 | 0.49 | 2.56 | 0.41 | 0.0 |
|  | Thakkar^[16](#_ENREF_16)^ | 2016 | SCZ | 12 | 21 | 2.55 | 0.49 | 2.32 | 0.45 | -0.5 |
| Striatum | Goto[^1^](#_ENREF_1)^,^ [^2^](#_ENREF_2) | 2009 | FEP | 18 | 18 | 1.73 | 0.81 | 1.12 | 0.71 | -0.8 |
|  | Tayoshi^[4](#_ENREF_4)^ | 2010 | SCZ | 29 | 38 | 1.18 | 0.20 | 1.13 | 0.26 | -0.2 |
|  | de la Fuente Sandoval[^8^](#_ENREF_8) | 2015 | CHR | 24 | 23 | 1.80^-3^ | 0.27^-3^ | 2.21^-3^ | 0.36^-3^ | 1.3 |
|  | Thakkar^[16](#_ENREF_16)^ | 2016 | Sibling | 12 | 23 | 2.36 | 0.70 | 2.16 | 0.62 | -0.3 |
|  | Thakkar^[16](#_ENREF_16)^ | 2016 | SCZ | 12 | 21 | 2.36 | 0.70 | 2.31 | 0.87 | -0.1 |
| dlPFC | Kegeles^[5](#_ENREF_5)^ | 2012 | SCZ unmed | 11 | 16 | 5.00 x 10^-3^ | 0.98 x 10^-3^ | 4.88 x 10^-3^ | 1.64 x 10^-3^ | -0.1 |
| dlPFC | Kegeles[^5^](#_ENREF_5) | 2012 | SCZ med | 11 | 16 | 5.00 x 10^-3^ | 0.98 x 10^-3^ | 4.65 x 10^-3^ | 0.83 x 10^-3^ | -0.4 |
| HC | Stan[^17^](#_ENREF_17) | 2015 | SCZ | 16 | 18 | 0.34 | 0.06 | 0.33 | 0.05 | -0.2 |
| CSO | Rowland[^6^](#_ENREF_6) | 2013 | SCZ young | 10 | 11 | 0.94 | 0.12 | 0.96 | 0.12 | 0.2 |
| CSO | Rowland[^6^](#_ENREF_6) | 2013 | SCZ old | 10 | 10 | 0.95 | 0.12 | 0.93 | 0.14 | -0.7 |

**Supplementary Table 1. ^1^H-MRS GABA Studies: Group means and standard deviations GABA level for calculation of effect size.**

Abbreviations: C: Control; CHR Clinical High Risk; CSO: centrum semiovale; dlPFC: dorsolateral prefrontal cortex; FEP: First Episode Psychosis; *g*: Hedge’s *g* effect size; HC: hippocampus; *P*: significance of group difference; mFC: medial frontal cortex; POC: parietal/occipital cortex; SCZ: Schizophrenia or schizophreniform disorder; Sibling: healthy siblings of patients with SCZ.

| **Region** | **First**  **Author** | **Year** | **Patient group** | **T** | **Vol.** | **Time** | **SNR** | **CRLB %**  **Max, c, scz** | ***g*** | **P** | **%CV** | | **GM%** | |
| --- | --- | --- | --- | --- | --- | --- | --- | --- | --- | --- | --- | --- | --- | --- |
|  |  |  |  |  |  |  |  |  |  |  | **C** | **SCZ** | **C** | **SCZ** |
| mFC | Goto[^1^](#_ENREF_1)^,^ [^2^](#_ENREF_2) | 2009 | FEP | 3 | 27 | 6 | 486 | NR | -0.2 | NS | 89 | 79 | NR | |
|  | Öngür^[3](#_ENREF_3)^ | 2010 | SCZ | 4 | 16.7 | 9 | 601 | 50, 23, 20 | 0.5 | NS | 31 | 25 | 62±5 | 61±5 |
|  | Tayoshi^[4](#_ENREF_4)^ | 2010 | SCZ | 3 | 27 | 13 | 1053 | 20, NR, NR | -0.3 | NS | 36 | 33 | 37±5 | 38±8 |
|  | Kegeles^[5](#_ENREF_5)^ | 2012 | SCZ unmed | 3 | 18.8 | 13 | 733 | NR | 0.7 | 0.02 | 15 | 36 | 51±2 | 51±2 |
|  | Kegeles^[5](#_ENREF_5)^ | 2012 | SCZ med | 3 | 18.8 | 13 | 733 | NR | 0.2 | NS | 15 | 44 | 51±2 | 50±5 |
|  | Rowland[^6^](#_ENREF_6) | 2013 | SCZ young | 3 | 42.9 | 8.8 | 1133 | NR | 0.0 | NS | 23 | 15 | 47±5 | 50±3 |
|  | Rowland[^6^](#_ENREF_6) | 2013 | SCZ old | 3 | 42.9 | 8.8 | 1133 | NR | -1.0 | 0.037 | 17 | 20 | 47±6 | 47±3 |
|  | Marsman^[7](#_ENREF_7)^ | 2014 | SCZ | 7 | 8 | 4 | 224 | 20, NR, NR | -0.8 | 0.012 | 21 | 17 | 68±11 | 68±10 |
|  | de la Fuente Sandoval[^8^](#_ENREF_8) | 2015 | CHR | 3 | 18.8 | 13.4 | 756 | NR | 0.7 | 0.03 | 15 | 16 | 52±3 | 53±4 |
|  | Brandt[^9^](#_ENREF_9) | 2016 | SCZ | 7 | 7.2 | 5.5 | 277 | 20, NR, NR | 0.3 | NS | 14 | 17 | NR | |
|  | Marenco^[10](#_ENREF_10)^ | 2016 | Sibling | 3 | 18 | 20.3 | 1096 | NR | -0.7 | <0.001 | 9 | 10 | 69±5 | 70±7 |
|  | Marenco^[10](#_ENREF_10)^ | 2016 | SCZ unmed | 3 | 18 | 20.3 | 1096 | NR | -0.4 | NS | 9 | 8 | 69±5 | 70±7 |
|  | Marenco^[10](#_ENREF_10)^ | 2016 | SCZ med | 3 | 18 | 20.3 | 1096 | NR | -0.3 | 0.02 | 9 | 10 | 69±5 | 69±6 |
|  | Menschikov^[11](#_ENREF_11)^ | 2016 | CHR | 3 | 27 | 8.8 | 24 | NR | -4.1 | 0.001 | 3 | 5 | NR | |
|  | Rowland[^12^](#_ENREF_12) | 2016 | SCZ young | 3 | 24 | 8.8 | 634 | 15, NR, NR | 0.0 | NS | 15 | 11 | NR | |
|  | Rowland[^12^](#_ENREF_12) | 2016 | SCZ old | 3 | 24 | 8.8 | 634 | 15, NR, NR | -0.7 | <0.005 | 19 | 19 | NR | |
|  | Yang[^13^](#_ENREF_13) | 2016 | FEP | 3 | 27 | 3.3 | 267 | NR | 0.8 | 0.012 | 17 | 24 | NR | |
| POC | Goto[^1^](#_ENREF_1)^,^ [^2^](#_ENREF_2) | 2009 | FEP | 3 | 27 | 6 | 486 | NR | 0.2 | NS | 59 | 46 | NR | |
|  | Öngür^[3](#_ENREF_3)^ | 2010 | SCZ | 4 | 16.7 | 9 | 601 | 50, 21, 21 | 0.4 | NS | 25 | 25 | 56±6 | 56±6 |
|  | Yoon[^14^](#_ENREF_14) | 2010 | FEP/SCZ | 3 | 26.25 | 6.5 | 512 | NR | -2.6 | <0.05 | 5 | 3 | NR | |
|  | Kelemen^[15](#_ENREF_15)^ | 2013 | FEP | 3 | 26.25 | 6.5 | 512 | NR | -0.7 | <0.05 | 27 | 63 | NR | |
|  | Marsman^[7](#_ENREF_7)^ | 2014 | SCZ | 7 | 8 | 4 | 224 | 20, NR, NR | 0.3 | NS | 21 | 27 | 68±10 | 69±8 |
|  | Thakkar^[16](#_ENREF_16)^ | 2016 | Sibling | 7 | 24 | 5.4 | 907 | 20, 3, 3 | 0.0 | NS | 19 | 19 | 62±3 | 63±3 |
|  | Thakkar^[16](#_ENREF_16)^ | 2016 | SCZ | 7 | 24 | 5.4 | 907 | 20, 3, 3 | -0.5 | NS | 19 | 16 | 62±3 | 60±4 |
| Striatum | Goto[^1^](#_ENREF_1)^,^ [^2^](#_ENREF_2) | 2009 | FEP | 3 | 27 | 6 | 486 | NR | -0.8 | 0.024 | 47 | 63 | NR | |
|  | Tayoshi^[4](#_ENREF_4)^ | 2010 | SCZ | 3 | 27 | 13 | 1053 | 20, NR, NR | -0.2 | NS | 17 | 23 | 35±1 | 34±1 |
|  | de la Fuente Sandoval[^8^](#_ENREF_8) | 2015 | CHR | 3 | 22.5 | 13.4 | 905 | NR | 1.3 | <0.001 | 15 | 16 | 35±4 | 36±5 |
|  | Thakkar^[16](#_ENREF_16)^ | 2016 | Sibling | 7 | 24 | 5.4 | 907 | 20, 4, 4 | -0.3 | NS | 30 | 38 | 52±3 | 51±5 |
|  | Thakkar^[16](#_ENREF_16)^ | 2016 | SCZ | 7 | 24 | 5.4 | 907 | 20, 4, 4 | -0.1 | NS | 30 | 28 | 52±3 | 50±4 |
| dlPFC | Kegeles^[5](#_ENREF_5)^ | 2012 | SCZ unmed | 3 | 9.6 | 26 | 748 | NR | -0.1 | NS | 20 | 34 | 52±6 | 51±6 |
| dlPFC | Kegeles^[5](#_ENREF_5)^ | 2012 | SCZ med | 3 | 9.6 | 26 | 748 | NR | -0.4 | NS | 20 | 18 | 52±6 | 50±5 |
| HC | Stan[^17^](#_ENREF_17) | 2015 | SCZ | 3 | 11.25 | 16 | 540 | NR, 6 | -0.2 | NS | 15 | 18 | NR | |
| CSO | Rowland[^6^](#_ENREF_6) | 2013 | SCZ young | 3 | 45 | 8.8 | 1188 | NR | 0.2 | NS | 13 | 13 | 21±3 | 19±3 |
| CSO | Rowland[^6^](#_ENREF_6) | 2013 | SCZ old | 3 | 45 | 8.8 | 1188 | NR | -0.7 | NS | 15 | 13 | 24±8 | 22±2 |

**Supplementary Table 2. ^1^H-MRS GABA Studies: Methodological Variables.**

Abbreviations: C: Control; CHR Clinical High Risk; CRLB: Cramer-Rao lower bound, maximum, mean in controls, mean in SCZ; CSO: centrum semiovale; dlPFC: dorsolateral prefrontal cortex; FEP: First Episode Psychosis; *g*: Hedge’s *g* effect size; HC: hippocampus; *P*: significance of group difference; %CV: percent coefficient of variation; %GM: percentage grey matter in voxel; mFC: medial frontal cortex; POC: parietal/occipital cortex; SCZ: Schizophrenia or schizophreniform disorder; Sibling: healthy siblings of patients with SCZ; SNR: signal to noise ratio (field strength x volume x √time); T: Field strength of MRI scanner in Tesla; Time: acquisition time in minutes; NS non-significant; NR: not reported; Vol: voxel volume in millilitres.

| **Author** | **Year** | **Patient group** | **Imaging** | **Radiotracer** | **Index** | **Reference tissue** | **Analysis** | **%CV** | |
| --- | --- | --- | --- | --- | --- | --- | --- | --- | --- |
|  |  |  |  |  |  |  |  | **C** | **SCZ** |
| Busatto^[18](#_ENREF_18)^ | 1997 | SCZ | SPECT | [^123^I] Iomazenil, | BP (ratio) | White matter plus ventricles | ROI | 21 | 18 |
| Verhoeff^[19](#_ENREF_19)^ | 1999 | SCZ | SPECT | [^123^I] Iomazenil | V_3_-p | NA | Voxel-wise | - | - |
| Abi-Dargham^[20](#_ENREF_20)^ | 1999 | SCZ | SPECT | [^123^I] Iomazenil, | V_T_ | NA | ROI, voxel-wise | 23 | 21 |
| Asai^[21](#_ENREF_21)^ | 2008 | SCZ | PET | [^11^C] Ro15-4513 | BP (SRTM) | Pons | ROI | 13 | 13 |
| Lee[^22^](#_ENREF_22) | 2013 | SCZ | PET | [^18^F] Fluoro-flumazenil | BP (MLRTM) | Pons | Voxel-wise | - | - |
| Kang[^23^](#_ENREF_23) | 2014 | CHR | PET | [^18^F] Fluoro-flumazenil | BP (MLRTM) | Pons | Voxel-wise, ROI | 6 | 4 |
| Frankle^[24](#_ENREF_24)^ | 2015 | SCZ | PET | [^11^C] Flumazenil | V_T_ | NA | ROI | 10 | 12 |

**Supplementary Table 3. GABA_A_/BDZ receptor availability radiotracer Studies: Methodological Variables.**

Abbreviations: BP binding potential; C: Control; CHR Clinical High Risk; MLRTM: multi-linear reference tissue model; %M percentage male in sample; NA: not applicable; PET: positron emission tomography; %CV: percent coefficient of variation, as the mean across all ROI reported in the article; ROI: region of interest; SCZ: Schizophrenia or schizophreniform disorder; SPECT: single photon emission computed tomography; SRTM simplified reference tissue model; V_3_-p: volume of distribution with normalisation for total brain uptake; V_T_ Volume of distribution.

**Study methodological characteristics.**

There are not established criteria for formally evaluation the quality of either ^1^H-MRS or PET/SPECT studies. To aid evaluation of the published GABA imaging studies in schizophrenia and provide recommendations for future studies, the section below provides discussion of the principal factors that may impact on data quality.

**^1^H-MRS GABA**

*Data acquisition methods*

All of the identified ^1^H-MRS GABA studies in schizophrenia used J-difference editing methods for GABA detection, with the exception of one study at 7 Tesla (T) which used a stimulated echo acquisition mode (STEAM) sequence.[^9^](#_ENREF_9) At higher field strengths, the signal to noise ratio (SNR) and ability to resolve metabolites increases (see [^25^](#_ENREF_25)). The majority of studies in schizophrenia acquired data at a field strength of 3T,[^1^](#_ENREF_1)^,^ [^2^](#_ENREF_2)^,^ [^4-6^](#_ENREF_4)^,^ [^8^](#_ENREF_8)^,^ [^10-15^](#_ENREF_10)^,^ [^17^](#_ENREF_17) one study acquired data at 4T[^3^](#_ENREF_3) and three studies acquired data at 7T[^7^](#_ENREF_7)^,^ [^9^](#_ENREF_9)^,^ [^16^](#_ENREF_16) (Supplement Table 2). A crude estimate of measurement quality as the SNR, which does not account for differences in acquisition protocols, can be calculated by multiplying the field strength (T) by the volume of the voxel (mL) by the square root of the acquisition time (minutes) (see [^26^](#_ENREF_26)). There is therefore a pay-off between maximising SNR and minimizing acquisition time and voxel size. Increased acquisition time may associate with greater subject discomfort and movement, and increased voxel size will decrease anatomical specificity.

Supplementary Table 2 shows the volume, acquisition time and the crude SNR estimate (field strength x volume x √time) for ^1^H-MRS GABA studies in schizophrenia. These studies have on average acquired data in voxel sizes of 30 ± 10 ml, in an acquisition time of 10 ± 6 minutes. Arbitrary identification (> 1 s.d. from the mean) indicates the particularly large voxels in the mFC and centrum semiovale in the article by Rowland et al., 2013 at 3T[^6^](#_ENREF_6), whereas two studies at 7T[^7^](#_ENREF_7)^,^ [^9^](#_ENREF_9) utilized the higher field strength to acquire data in smaller, and therefore more anatomically specific voxels. Relatively long acquisition times (> 1 s.d. from the mean) were used to acquire data in the medial frontal cortex in Marenco et al.,[^10^](#_ENREF_10) and in the dorsolateral prefrontal cortex in Kegeles et al.,[^5^](#_ENREF_5) whereas the 7T study by Marsman et al.,[^7^](#_ENREF_7) utilized a relatively short acquisition time (4 min) which may have minimized subject movement. The study by Menschikov et al.,^[11](#_ENREF_11)^ used a very short acquisition time of 16 seconds. As this study was performed at the lower field strength of 3T, the associated SNR is particularly low (SNR of 24 compared to a mean ± s.d. SNR of 729 ± 316 across studies and regions), which may have resulted in poor data quality. Also at 3T, the SNR was low (> 1 s.d. from the mean) in the study by Yang et al.,.^[13](#_ENREF_13)^ In contrast, three studies[^4^](#_ENREF_4)^,^ [^6^](#_ENREF_6)^,^ [^10^](#_ENREF_10) were associated with relatively high SNRs.

In addition, the ^1^H-MRS GABA signal is subject to contamination by macromolecules due to overlapping resonances (see [^27^](#_ENREF_27)), which can therefore be denoted as GABA+. There are emerging approaches to correct for this, and of the articles included in this review only two recent studies employed techniques to eliminate macromolecules from the GABA signal.[^12^](#_ENREF_12)^,^ [^16^](#_ENREF_16)

*Data quality control.*

Cramér Rao lower bounds (CRLB) are used to express the reliability of metabolite concentration estimates in ^1^H-MRS studies, and for quality control procedures a cut off of CRLB >20% is commonly accepted as an exclusion criteria for poor quality spectra. However CRLB do not reflect all sources of error and relying solely on CRLB cut-offs for exclusion may introduce bias.[^28^](#_ENREF_28) The use of CRLB may be particularly problematic where a disorder is suspected to be associated with a reduction in the metabolite to near-noise level.[^28^](#_ENREF_28) This may apply to investigation of GABA in schizophrenia, as GABA concentrations are low compared to other commonly measured metabolites, and as further GABA reduction may be hypothesised to occur in the patient group. The optimal approach may therefore be visual inspection of individual spectra for quality control (which are not available in published articles) by an expert blind to group allocation, and reporting of the CRLB in the patient and control groups.

Of the sixteen ^1^H-MRS studies in schizophrenia, ten did not provide information on using CRLB for exclusion of poor quality spectra or report the achieved CRLB in the control and patient groups (Supplementary Table 1,[^1^](#_ENREF_1)^,^ [^2^](#_ENREF_2)^,^ [^5^](#_ENREF_5)^,^ [^6^](#_ENREF_6)^,^ [^8^](#_ENREF_8)^,^ [^10^](#_ENREF_10)^,^ [^11^](#_ENREF_11)^,^ [^13-15^](#_ENREF_13)). However, two[^8^](#_ENREF_8)^,^ [^10^](#_ENREF_10) of these ten studies provided detailed information on procedures for spectral quality assessment procedures. Of the studies that did apply a CRLB threshold for inclusion, this ranged from CRLB associated with the GABA estimate of <15%,[^12^](#_ENREF_12) <20%[^4^](#_ENREF_4)^,^ [^7^](#_ENREF_7)^,^ [^9^](#_ENREF_9)^,^ [^16^](#_ENREF_16) to < 50%.[^3^](#_ENREF_3) On the basis of either providing detailed spectra quality control procedures or data with CRLB of <20%, data might be judged of good quality in eight articles,[^4^](#_ENREF_4)^,^ [^7-10^](#_ENREF_7)^,^ [^12^](#_ENREF_12)^,^ [^16^](#_ENREF_16)^,^ [^17^](#_ENREF_17), of moderate quality in one article[^3^](#_ENREF_3) and of unknown quality in seven articles.[^1^](#_ENREF_1)^,^ [^2^](#_ENREF_2)^,^ [^5^](#_ENREF_5)^,^ [^6^](#_ENREF_6)^,^ [^11^](#_ENREF_11)^,^ [^13-15^](#_ENREF_13)

*Between-subjects variability*

To evaluate the variability of the measurement as an indicator of reliability, Supplementary Table 2 also provides the coefficient of variation (CV; standard deviation / mean) for the reported ^1^H-MRS GABA measurement in the control and patient groups. Studies with high CVs will be less likely to detect significant effects, therefore studies with high CVs and non-significant results may not have detected differences because of variability in the measurement, whereas studies with high CVs and significant results may reflect spurious findings. Across all studies and regions, the variability in control (22 ± 16%) and patient (25 ± 17%) groups was similar, suggesting that clinical, genetic or other variability (for example decreased tolerance of the scanning procedure) that may be associated with patient samples did not systematically associate with increased variability in GABA measurement in patients compared to control. Compared to other studies, data in the study of Goto et al.,[^1^](#_ENREF_1)^,^ [^2^](#_ENREF_2) showed high variability across all voxels investigated (>1 s.d. from the mean for the control group), while variability in the studies of Menschikov et al.,[^11^](#_ENREF_11) and Yoon et al.,[^14^](#_ENREF_14) was low.

**PET/SPECT studies.**

All PET/SPECT used radiotracers with affinity for the BDZ binding site on the GABA_A_/BZR complex (Supplement Table 3). The first three published articles acquired data with [^123^I]iomazenil SPECT,[^18-20^](#_ENREF_18) while the four more recently published articles acquired data using the higher spatial resolution approach of PET imaging.[^21-24^](#_ENREF_21) Of the PET studies, three employed the GABA_A_/BDZ receptor antagonist flumazenil, as [^18^F]fluoro-flumazenil[^22^](#_ENREF_22)^,^ [^23^](#_ENREF_23) or as [^11^C]flumazenil[^24^](#_ENREF_24) which has a shorter half-life. One PET study employed the [^11^C]Ro15-4513, [^21^](#_ENREF_21) which has greater selectivity for GABA_A_/BDZ receptors containing α1 and α5 subunits.[^29^](#_ENREF_29)^,^ [^30^](#_ENREF_30)

Regional GABA_A_/BDZ receptor availability was estimated either as the binding potential (BP), relative to the signal in a reference tissue representing ‘non-specific binding’,[^18^](#_ENREF_18)^,^ [^21-23^](#_ENREF_21) or as the regional distribution volume (V_T_), to avoid potential influences of non-negligible specific binding in the reference tissue.[^19^](#_ENREF_19)^,^ [^20^](#_ENREF_20)^,^ [^24^](#_ENREF_24) The SPECT study which estimated BP used a semi-quantitative method to estimate the binding ratio between the ROI and the reference tissue (white matter and ventricles).[^18^](#_ENREF_18) The more recent PET studies estimated BP in the ROI relative to the pons reference tissue, using more sophisticated kinetic modelling approaches. [^21-23^](#_ENREF_21) Estimation of V_T_ in the remaining studies avoided the use of a reference tissue through the use of a plasma radioactivity input function for kinetic modelling. [^19^](#_ENREF_19)^,^ [^20^](#_ENREF_20)^,^ [^24^](#_ENREF_24)

Four articles reported region of interest (ROI) analysis primarily,[^18^](#_ENREF_18)^,^ [^20^](#_ENREF_20)^,^ [^21^](#_ENREF_21)^,^ [^24^](#_ENREF_24) two articles reported voxel-wise (whole brain) analyses to avoid *a priori* hypotheses about specific ROI [^19^](#_ENREF_19)^,^ [^22^](#_ENREF_22) and one article reported voxel-wise analysis with secondary ROI analyses of significant clusters.[^23^](#_ENREF_23)

The overall variability (CV) in primary outcome measures of GABA_A_/BDZ receptor availability, across all regions and studies, was 19 ± 7% in the control group and 14 ± 6% in the patient group. Therefore, as for ^1^H-MRS studies, this also does not suggest more variability in the clinical group.

**References**

**1.** Goto N, Yoshimura R, Kakeda S, et al. No alterations of brain GABA after 6 months of treatment with atypical antipsychotic drugs in early-stage first-episode schizophrenia. *Prog Neuropsychopharmacol Biol Psychiatry* Dec 01 2010;34(8):1480-1483.

**2.** Goto N, Yoshimura R, Moriya J, et al. Reduction of brain gamma-aminobutyric acid (GABA) concentrations in early-stage schizophrenia patients: 3T Proton MRS study. *SchizophrRes* 2009;112(1-3).

**3.** Ongur D, Prescot AP, McCarthy J, Cohen BM, Renshaw PF. Elevated gamma-aminobutyric acid levels in chronic schizophrenia. *Biol Psychiatry* 2010;68(7):667-670.

**4.** Tayoshi S, Nakataki M, Sumitani S, et al. GABA concentration in schizophrenia patients and the effects of antipsychotic medication: a proton magnetic resonance spectroscopy study. *SchizophrRes* 2010;117(1):83-91.

**5.** Kegeles LS, Mao X, Stanford AD, et al. Elevated prefrontal cortex gamma-aminobutyric acid and glutamate-glutamine levels in schizophrenia measured in vivo with proton magnetic resonance spectroscopy. *ArchGenPsychiatry* 2012;69(5):449-459.

**6.** Rowland LM, Kontson K, West J, Edden RA, Zhu H, Wijtenburg SA, Holcomb HH, Barker PB. In vivo measurements of glutamate, GABA, and NAAG in schizophrenia. *Schizophr Bull* Sep 2013;39(5):1096-1104.

**7.** Marsman A, Mandl RC, Klomp DW, et al. GABA and glutamate in schizophrenia: A 7 T (1)H-MRS study. *NeuroImage Clinical* 2014;6:398-407.

**8.** de la Fuente-Sandoval C, Reyes-Madrigal F, Mao X, et al. Cortico-Striatal GABAergic and Glutamatergic Dysregulations in Subjects at Ultra-High Risk for Psychosis Investigated with Proton Magnetic Resonance Spectroscopy. *The international journal of neuropsychopharmacology* Sep 12 2015;19(3):pyv105.

**9.** Brandt AS, Unschuld PG, Pradhan S, et al. Age-related changes in anterior cingulate cortex glutamate in schizophrenia: A (1)H MRS Study at 7 Tesla. *Schizophr Res* Apr 2016;172(1-3):101-105.

**10.** Marenco S, Meyer C, Kuo S, et al. Prefrontal GABA Levels Measured With Magnetic Resonance Spectroscopy in Patients With Psychosis and Unaffected Siblings. *Am J Psychiatry* May 01 2016;173(5):527-534.

**11.** Menschikov PE, Semenova NA, Ublinskiy MV, et al. (1)H-MRS and MEGA-PRESS pulse sequence in the study of balance of inhibitory and excitatory neurotransmitters in the human brain of ultra-high risk of schizophrenia patients. *Doklady Biochemistry and biophysics* May 2016;468(1):168-172.

**12.** Rowland LM, Krause BW, Wijtenburg SA, et al. Medial frontal GABA is lower in older schizophrenia: a MEGA-PRESS with macromolecule suppression study. *Mol Psychiatry* Feb 2016;21(2):198-204.

**13.** Yang Z, Zhu Y, Song Z, et al. Comparison of the density of gamma-aminobutyric acid in the ventromedial prefrontal cortex of patients with first-episode psychosis and healthy controls. *Shanghai archives of psychiatry* Dec 25 2015;27(6):341-347.

**14.** Yoon JH, Maddock RJ, Rokem A, Silver MA, Minzenberg MJ, Ragland JD, Carter CS. GABA concentration is reduced in visual cortex in schizophrenia and correlates with orientation-specific surround suppression. *JNeurosci* 2010;30(10):3777-3781.

**15.** Kelemen O, Kiss I, Benedek G, Keri S. Perceptual and cognitive effects of antipsychotics in first-episode schizophrenia: the potential impact of GABA concentration in the visual cortex. *Prog Neuropsychopharmacol Biol Psychiatry* Dec 02 2013;47:13-19.

**16.** Thakkar KN, Rosler L, Wijnen JP, Boer VO, Klomp DW, Cahn W, Kahn RS, Neggers SF. 7T Proton Magnetic Resonance Spectroscopy of Gamma-Aminobutyric Acid, Glutamate, and Glutamine Reveals Altered Concentrations in Patients With Schizophrenia and Healthy Siblings. *Biol Psychiatry* Apr 19 2016.

**17.** Stan AD, Ghose S, Zhao C, et al. Magnetic resonance spectroscopy and tissue protein concentrations together suggest lower glutamate signaling in dentate gyrus in schizophrenia. *Mol Psychiatry* Apr 2015;20(4):433-439.

**18.** Busatto GF, Pilowsky LS, Costa DC, Ell PJ, David AS, Lucey JV, Kerwin RW. Correlation between reduced in vivo benzodiazepine receptor binding and severity of psychotic symptoms in schizophrenia. *Am J Psychiatry* Jan 1997;154(1):56-63.

**19.** Verhoeff NP, Soares JC, D'Souza CD, et al. [123I]Iomazenil SPECT benzodiazepine receptor imaging in schizophrenia. *Psychiatry Res* Oct 11 1999;91(3):163-173.

**20.** Abi-Dargham A, Laruelle M, Krystal J, et al. No evidence of altered in vivo benzodiazepine receptor binding in schizophrenia. *Neuropsychopharmacology* 1999;20(6).

**21.** Asai Y, Takano A, Ito H, et al. GABAA/Benzodiazepine receptor binding in patients with schizophrenia using [11C]Ro15-4513, a radioligand with relatively high affinity for alpha5 subunit. *SchizophrRes* 2008;99(1-3).

**22.** Lee JS, Lee JD, Park HJ, Oh MK, Chun JW, Kim SJ, Kim E, Kim JJ. Is the GABA System Related to the Social Competence Improvement Effect of Aripiprazole? An (18)F-Fluoroflumazenil PET Study. *Psychiatry Investig* Mar 2013;10(1):75-80.

**23.** Kang JI, Park HJ, Kim SJ, et al. Reduced binding potential of GABA-A/benzodiazepine receptors in individuals at ultra-high risk for psychosis: an [18F]-fluoroflumazenil positron emission tomography study. *Schizophr Bull* May 2014;40(3):548-557.

**24.** Frankle WG, Cho RY, Prasad KM, et al. In vivo measurement of GABA transmission in healthy subjects and schizophrenia patients. *Am J Psychiatry* Nov 01 2015;172(11):1148-1159.

**25.** Tkac I, Gruetter R. Methodology of H NMR Spectroscopy of the Human Brain at Very High Magnetic Fields. *Applied magnetic resonance* Mar 2005;29(1):139-157.

**26.** Puts NA, Edden RA. In vivo magnetic resonance spectroscopy of GABA: a methodological review. *Progress in nuclear magnetic resonance spectroscopy* Jan 2012;60:29-41.

**27.** Mullins PG, McGonigle DJ, O'Gorman RL, Puts NA, Vidyasagar R, Evans CJ, Cardiff Symposium on MRSoG, Edden RA. Current practice in the use of MEGA-PRESS spectroscopy for the detection of GABA. *Neuroimage* Feb 01 2014;86:43-52.

**28.** Kreis R. The trouble with quality filtering based on relative Cramer-Rao lower bounds. *Magn Reson Med* Jan 2016;75(1):15-18.

**29.** Lingford-Hughes A, Hume SP, Feeney A, et al. Imaging the GABA-benzodiazepine receptor subtype containing the alpha5-subunit in vivo with [11C]Ro15 4513 positron emission tomography. *J Cereb Blood Flow Metab* Jul 2002;22(7):878-889.

**30.** Luddens H, Seeburg PH, Korpi ER. Impact of beta and gamma variants on ligand-binding properties of gamma-aminobutyric acid type A receptors. *Molecular pharmacology* May 1994;45(5):810-814.
